# Translational Relevance of SCA1 Models for the Development of Therapies for Spinocerebellar Ataxia Type 1

**DOI:** 10.3390/biomedicines13123066

**Published:** 2025-12-12

**Authors:** Elizaveta Plotnikova, Tatyana Ageeva, Albert Sufianov, Galina Sufianova, Albert Rizvanov, Yana Mukhamedshina

**Affiliations:** 1 OpenLab Gene and Cell Technologies, Institute of Fundamental Medicine and Biology, Kazan Federal University, 420008 Kazan, Russiarizvanov@gmail.com (A.R.); yana.k-z-n@mail.ru (Y.M.); 2 Federal State Budgetary Scientific Institution “Russian Scientific Center of Surgery Named After Academician B.V. Petrovsky”, 119991 Moscow, Russia; 3 The Research and Educational Institute of Neurosurgery, Peoples’ Friendship University of Russia (RUDN), 117198 Moscow, Russia; 4 Department of Pharmacology, Tyumen State Medical University, 625023 Tyumen, Russia; 5 Division of Medical and Biological Sciences, Tatarstan Academy of Sciences, 420111 Kazan, Russia; 6 Department of Histology, Cytology and Embryology, Kazan State Medical University, 420012 Kazan, Russia

**Keywords:** SCA1, ATXN1, polyglutamine expansion, Purkinje neurons, ATXN1-CIC, 14-3-3, B05, HEK293, translational medicine, targeted therapy

## Abstract

Spinocerebellar ataxia type 1 (SCA1) is an autosomal dominant neurodegenerative dis-ease caused by the expansion of cytosine–adenine–guanine (CAG) repeats in the ataxin-1 (*ATXN1*) gene, leading to toxic gain-of-function of the ataxin-1 (ATXN1) protein. This narrative review systematizes the clinical and genetic aspects of SCA1 and discusses key molecular and cellular mechanisms: the ATXN1-CIC ataxin-1-Capicua complex (ATXN1-CIC), the role of serine 776 (Ser776) phosphorylation, interactions with 14-3-3 proteins, transcriptional dysregulation, and critically analyzes experimental models of the disease in vivo and in vitro. In addition, it presents a descriptive quantitative analysis of the literature on in vivo SCA1 models, conducted using a defined search methodology with a cut-off date of 23 November 2025. For each model, phenotypic markers, molecular signatures, and applicability to preclinical testing tasks are summarized. A comparison of the models reveals their complementarity and outlines optimal research trajectories, including omics approaches and prospects for targeted antisense oligonucleotide (ASO) therapy, RNA interference (RNAi), and genome editing. The result is a practical guide for selecting a model in accordance with specific hypotheses and translational objectives.

## 1. Introduction

Spinocerebellar ataxia type 1 (SCA1) is a prototypical disease from the group of polyglutamine neurodegenerations, arising from the expansion of CAG repeats in the *ATXN1* gene [1,2]. Despite its low prevalence (1–2 cases per 100,000 population), SCA1 attracts attention as a model for studying the toxic gain-of-function mechanism characteristic of triplet diseases. The mutant ataxin-1 protein with an elongated polyglutamine track forms intranuclear inclusions, disrupts transcriptional networks, and triggers progressive degeneration of Purkinje cells and brain stem structures [3].

Longitudinal multimodal studies in patients with SCA1 have demonstrated a marked reduction in the volume of posterior fossa structures. Total brainstem and cerebellar volume, as well as the volumes of the pons, cerebellar cortex and cerebellar white matter, are reduced, and these measures show a strong negative correlation with ataxia severity as assessed by the SARA score [4,5]. Neuropathological data indicate that these changes reflect a combination of neuronal loss in the primary motor cortex with widespread degeneration of the grey matter of the basal forebrain, thalamus, brainstem and cerebellum, as well as the white matter of the cerebellum and brainstem [6]. The degenerative process also extends to other brainstem structures, including the red nucleus, vestibular nuclei and motor cranial nerve nuclei [7]. Furthermore, recent studies have demonstrated reduced numbers of oligodendrocytes in the cerebellar white matter in patients with SCA1, underscoring the contribution of myelinating glia to disease pathogenesis [8].

Clinically, SCA1 manifests as progressive cerebellar ataxia, dysarthria, and cognitive impairment, but the disease is multisystemic in nature. The earliest neurological manifestations typically include incoordination, such as gait disturbance with altered step length and unstable turning [9]. At later disease stages, a subset of patients may develop additional extrapyramidal features, including dystonia, tremor or choreiform hyperkinesias [10]. Muscle atrophy and fasciculations are also observed, indicating involvement of peripheral motor neurons. Oculomotor abnormalities are characteristic: nystagmus is present in approximately 25% of patients, whereas slow or hypermetric saccades are observed in about half of affected individuals [11]. In patients with SCA1 who have a moderate or severe disease course, pyramidal signs are frequently detected, including increased muscle tone and exaggerated tendon reflexes [12]. Late clinical manifestations include bulbar dysfunction due to brainstem involvement, typically emerging 10–15 years after disease onset and leading to impaired swallowing and respiration [13,14].

Cognitive deficits generally appear after the onset of motor symptoms and are predominantly characterized by mild impairment of executive functions and verbal memory [15]. Longitudinal studies [16,17] have shown that SCA1 progresses more rapidly than other forms of ataxia. In the EUROSCA cohort, annual worsening of ataxia (SARA score) was greatest in SCA1, intermediate in SCA2 and SCA3, and slowest in SCA6. Clinical manifestation occurs when there are 39–82 CAG repeats in ATXN1. In human patients, more than 39 uninterrupted polyQ repeats cause disease, and the longest repeat length reported in a human is 82Q, in a patient with juvenile SCA1, which is more severe and rapidly progressive [18]. The severity of symptoms and the rate of progression correlate positively with the length of the polyglutamine expansion [19]. Neuroimaging reveals cerebellar and brainstem atrophy even before clinical onset [20], with changes in pons and striatum volume reliably predicting motor deterioration [21]. Fluid biomarkers, including neurofilament light protein (NfL) levels, increase years before symptoms appear [22], opening up opportunities for early diagnosis and patient stratification.

A key pathogenic factor is the toxic effect of mutant ATXN1: ATXN1 knockout in mice does not cause an ataxic phenotype but leads to mild cognitive impairment [23], indicating an excess acquisition of pathological function rather than a simple loss of normal protein activity. Ser776 phosphorylation, stabilization of interactions with Capicua (CIC) and 14-3-3, and transcriptional control abnormalities play a central role [24,25]. However, the cascade of events linking CAG expansion to the selective vulnerability of Purkinje cells remains unclear.

A comparison of experimental models—from transgenic mice to induced pluripotent stem cell (iPSC) systems—has shown that each reproduces only certain aspects of the pathology [26,27]. A critical analysis of their strengths and weaknesses is necessary to select an adequate preclinical platform. This review summarizes the clinical and molecular features of SCA1, assesses the translational relevance of available models, and outlines the prospects for targeted approaches—from antisense oligonucleotides and RNAi to genome editing technologies.

## 2. Methods

**Figure 1:**
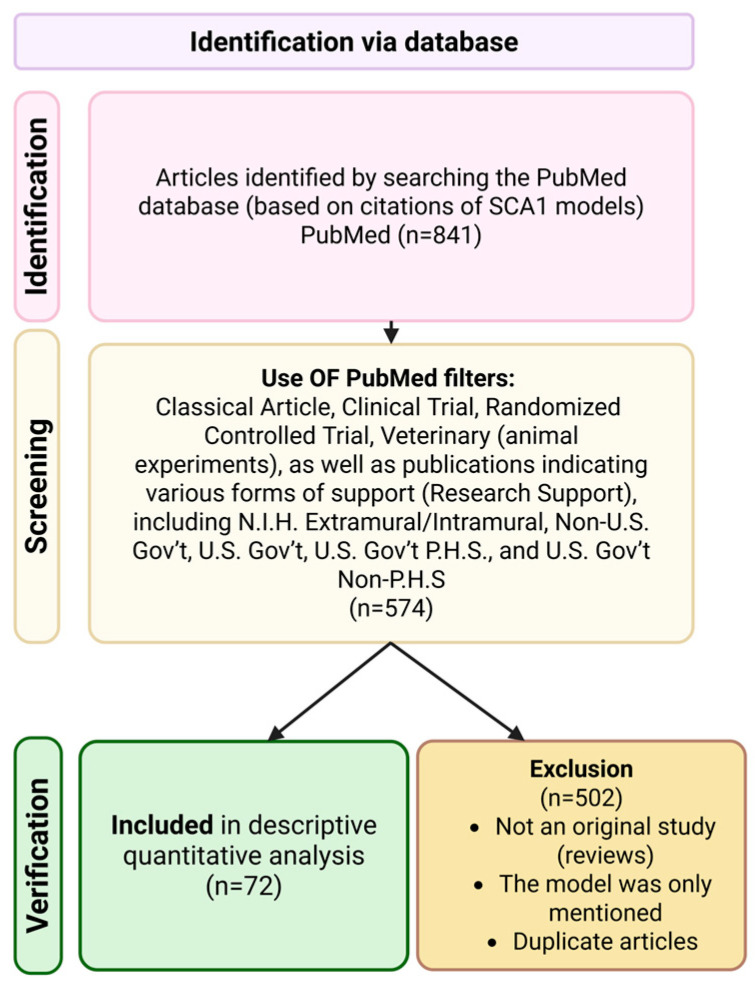
Flow diagram of the study selection process. The schematic illustrates the identification, screening, and inclusion process for publications analyzing SCA1 in vivo models. Initial search in PubMed database identified 841 records citing original SCA1 model publications. After applying publication type and research support filters, 574 records remained for screening. During eligibility assessment, 502 records were excluded due to not being original studies, only mentioning models without experimental use, or being duplicate publications. Finally, 72 studies met all criteria and were included in the descriptive quantitative analysis of SCA1 model utilization patterns.

To quantitatively assess the research utilization of established in vivo models of SCA1, we performed a structured analysis of the scientific literature indexed in PubMed. For each model3 = the original publication describing its generation was treated as the primary source, and citation of this paper was used as a proxy for subsequent experimental use of the model in original research. Importantly, only studies in which the model was explicitly employed as the experimental object were included in the analysis. Searches were executed individually for every model using the PubMed “Cited By” function. To restrict results to primary experimental studies, the following PubMed filters were applied: Article types—Classical Article, Clinical Trial, Randomized Controlled Trial, Veterinary (animal experiments); Research support categories—Research Support, N.I.H. Extramural, Research Support, N.I.H. Intramural, Research Support, Non-U.S. Gov’t, Research Support, U.S. Gov’t, Research Support, U.S. Gov’t, P.H.S., and Research Support, U.S. Gov’t, Non-P.H.S. The inclusion criteria encompassed only original research articles that used the referenced SCA1 model as the experimental object, including studies that made use of publicly available datasets generated using the respective SCA1 model. The exclusion criteria comprised: articles that cited the model’s original publication but did not use the model experimentally, review articles, and duplicate records. The original research articles included in the quantitative analysis are listed in Table 1. As summarized in Table 1, the most frequently used model was the knock-in ATXN1^[154Q/2Q]^ mouse line, which was identified in 31 eligible original research articles, followed by the transgenic ATXN1^[82Q]^ (B05) mouse model. A PRISMA-style flow diagram summarizing identification, screening, eligibility, and inclusion is provided in Figure 1. The date of the last search was 23 November 2025.

## 3. Chapter 1: Pathogenesis of SCA1

The human ATXN1 protein consists of 816 amino acids (~98–99 kDa) and is widely expressed in the brain, including the cerebellum and brainstem [85]. In SCA1, the toxicity of the expanded protein is a key factor, but recent data show that wild-type *ATXN1* performs critical physiological functions. In particular, in a model of autoimmune encephalomyelitis, partial loss of ATXN1 enhances demyelination, oligodendrocyte death, inflammation, and neurological deficits [79]. Thus, the pathogenesis of SCA1 is determined not only by toxic gain-of-function, but also by an imbalance between pathological and normal functions of ATXN1.

Much of the current mechanistic insight into SCA1 pathogenesis is based on data obtained from transgenic animal models and cellular systems. At the cellular level, the ATXN1 protein is localized in cerebellar and brainstem neurons predominantly in the nucleus, where it is part of large protein complexes on chromatin [86], whereas in non-specific tissues, it is found in the cytoplasm [87]. The development of SCA1 pathogenesis depends on the nuclear localization of mutant ATXN1, as various mutations in the C-terminal nuclear localization signal (NLS) disrupt its transport to the nucleus, which reduces protein toxicity [88]. Mutant ATXN1 protein can exist in the cytoplasm or nucleus of neurons either as soluble monomers/oligomers or as insoluble aggregates [89]. Phosphorylation of Ser 776 and binding to 14-3-3 proteins stabilize soluble mutant ATXN1 protein in the cytosol and protect it from immediate proteasomal degradation. Over time, the ATXN1–14-3-3 complex dissociates, and phosphorylated ATXN1 moves to the nucleus, where it can form insoluble aggregates [90]. In the event of phosphorylation disruption or 14-3-3 binding blockade, it accelerates proteasomal degradation of ataxin-1 in the cytoplasm and prevents nuclear migration. The main pathway for the removal of soluble cytoplasmic ataxin-1 is macroautophagy, but insoluble nuclear inclusions inhibit mTOR kinase, suppressing the late stages of autophagy and enhancing the accumulation of toxic forms of the protein [91].

Intranuclear aggregates of mutant ATXN1 disrupt proteostasis and protein synthesis by binding proteins to proteasomes, ribosomal subunits, ubiquitin ligases, kinases, and chaperones [92], suppressing the function of the ubiquitin–proteasome system (UPS) in the nucleus and enhancing cytotoxicity [91]. RNA transcripts accumulate in aggregates, contributing to ribosome dysfunction and proteome destabilization, exacerbating SCA1 pathology [93]. Nuclear inclusions show signs of phase separation, disrupt nucleocytoplasmic transport, and are involved in the disruption of the protein quality control system in SCA1 models [94]. In the ATXN1^[154Q/2Q]^ SCA1 model, it has been shown that Purkinje cells begin to form inclusions significantly later than the onset of functional impairment and morphological degeneration [95]. Furthermore, later studies have shown that nuclear aggregates mainly accumulate in areas of the brain that are not subject to degeneration, in contrast to Purkinje cells in the cerebellum, which are the first to lose viability [96].

Soluble oligomeric forms of mutant ATXN1, whose toxicity is mediated by interaction with the CIC cofactor in the nucleus, are key effectors of pathogenesis [61,97]. Thus, the insoluble (aggregated) form of ATXN1 is a consequence of the overload of cellular degradation systems and contributes to cellular dysfunction, but the most dangerous is considered to be the intermediate, soluble form of mutant ATXN1, which is able to move freely and interfere with nuclear processes. Zhang et al., 2020 [88] used proteomic analysis to study the interactome of mutant ATXN1^[85Q]^ in Neuro-2a neuroblastoma cells and identified 675 partner proteins, including key components of RAN signaling and nucleocytoplasmic transport factors. The authors confirmed that protein transport between the nucleus and cytoplasm is impaired both in cell culture and in Purkinje cells of a transgenic mouse model of SCA1 ATXN1^[82Q]^.

Similar processes are observed in other diseases associated with polyQ expansion. For example, in Huntington’s disease, mutant huntingtin aggregates trap important nuclear pore components (*Nup62*, *Nup88*) and RNA transport factors (*GLE1*, *RanGAP1*), leading to disruptions in nuclear-cytoplasmic exchange [98]. This suggests that impaired transport between the nucleus and cytoplasm may be a universal mechanism underlying a number of polyglutamine neurodegenerative pathologies. Qi et al. [99] showed that High-Mobility Group Box ½ (HMGB1/2) proteins are able to interact directly with mutant ataxin-1, leading to their retention in aggregates or removal from chromatin. Overexpression of HMGB1/2 proteins restored the ability to repair mitochondrial DNA damage, which was accompanied by the rescue of degenerating Purkinje cell dendrites; it weakened the activation of genotoxic signaling pathways (decreased γH2AX and p53 phosphorylation) [99,100]. Lam et al. [24] showed that SCA1 neuropathology depends on native, rather than new, ATXN1 interactions. In addition to Cic, ATXN1 interacts with nuclear co-repressors SMRT/HDAC3 [101], leucine-rich acidic nuclear protein (LANP) [38], histone deacetylase HDAC3, and other proteins.

Post-translational modifications such as phosphorylation of S776 and S239, ubiquitination, sumoylation, and proteolytic cleavage play an important role in the development of SCA1 pathology [43,102]. In a study by Vagiona et al. [103], 13 proteins involved in post-translational modifications of ATXN1 were identified, which differ in their expression levels in SCA1 model cells. In addition, mechanisms of negative regulation of ATXN1 were discovered at the RNA level: for example, microRNA miR-760 binds to an evolutionarily conserved site in the 5′-untranslated region of ATXN1 mRNA and induces degradation of this mRNA, suppressing the synthesis of both normal and mutant proteins [56]. Inhibition of miR-760, on the other hand, increases ATXN1 levels, confirming the importance of this regulatory mechanism.

14-3-3 proteins bind to phosphorylated ATXN1, keeping it in the nucleus and preventing aggregation and spontaneous dimerization, thereby directly affecting protein toxicity and stability [90,104]. S776 phosphorylation by cAMP-dependent protein kinase (PKA) regulates ATXN1 accumulation in the nucleus and its stability, enhancing protein toxicity [47,105]. In contrast, the Ser776Ala mutation reduces the level of mutant ATXN1 and alleviates disease symptoms in mice [71,106]. Interactions with other proteins, such as the splicing factors RNA-Binding Motif Protein 17 (RBM17) and U2 snRNP auxiliary factor 65 kDa (U2AF65), also depend on this phosphorylation [107]. De Chiara et al. [104] also showed that phosphorylation at S776 acts as a molecular switch that alters ATXN1interactions: the phosphorylated protein binds to 14-3-3, while the unphosphorylated protein binds to splicing factors such as U2AF65.

Lee et al. [108] showed that the phosphorylation site that affects mutant ATXN1 levels can be modulated by different regulators in selected brain regions. In the cerebellum, Ser776 phosphorylation is catalyzed by MSK1 kinase, while in the brainstem, RSK3 kinase is responsible for it. Combined inhibition of both kinases showed an additive effect, simultaneously improving coordination in both the cerebellum and brainstem of the SCA1 mouse model.

Another less studied ATXN1 phosphorylation site is Ser239 [109]. A review by Ju et al. [110] mentions that Ser239 phosphorylation is catalyzed by Nemo-like kinase (NLK) and that its alteration enhances ATXN1 toxicity in a Drosophila model.

In SCA1, early activation of the interferon pathway in Purkinje neurons from iPSCs of patients induces interferon-stimulated gene 15 (ISG15) expression. This protein, structurally similar to ubiquitin, disrupts the degradation of mutant ATXN1, contributing to its pathological accumulation in cells [36].

A key element of ATXN1 toxicity is the alteration of its protein interactions mediated by the AXH (Ataxin-1/HBP1) domain, which is sensitive to conformational changes during polyQ expansion [2,25,105]. The AXH domain is a highly conserved region of approximately 100 amino acids. Through the AXH domain, ATXN1 is capable of both homo-oligomerization and binding to a number of partners, including the transcription repressors CIC and growth factor independence 1 (GFI1) [24,111], Silencing Mediator of Retinoid and Thyroid Receptors (SMRT), and the retinoid-related orphan receptor α–tat-interacting protein 60 (RORα–Tip60) nuclear receptor complex.

The study by Pérez et al. [41] demonstrated that mutant ATXN1 has a dual effect on CIC transcriptional activity: on the one hand, it enhances its repressive function, leading to hyperrepression of target genes (gain-of-function mechanism), and on the other hand, it can contribute to a partial loss of CIC repressive activity, which highlights the complexity of the pathogenic effects of ATXN1. Among these genes are *Nrxn1*, *Cntn4*, *Cntn5*, *Lrp1b*, *Nrg3*, and others, whose products are necessary for the synaptic functions of Purkinje cells [25,112]. Mutations in the AXH domain that disrupt ATXN1–CIC interaction (e.g., at Val591 and Ser602) significantly mitigate molecular changes and the behavioral phenotype of SCA1 [66]. Viral delivery of an extra copy of the CIC gene using AAV into the degeneration-resistant caudal regions of the cerebellum showed that artificial overexpression of CIC was sufficient to induce neurodegeneration, confirming the key pathogenic role of CIC in the SCA1 model [54].

Normally, ATXN1 forms a triple complex with tat-interacting protein 60 (Tip60) and retinoid-related orphan receptor α (RORα) via its AXH domain. However, although mutant ATXN1 retains its ability to bind to Tip60, it cannot form a complete triple complex with RORα [103]. Mutant ATXN1 “displaces” the truncated isoform of the coactivator YAP (YAP_ΔC) from the complex with RORα, taking its place and thereby suppressing RORα-dependent transcription. A partial reduction in Tip60 levels in ATXN1^[82Q]^ mice slowed cerebellar neurodegeneration in the middle stages of the disease [113]. This results in a decrease in the transcription of RORα target genes necessary for the differentiation and maintenance of Purkinje cells. Staggerer mice, which carry a mutation in the RORα gene, develop a pronounced ataxic phenotype and cerebellar neuron degeneration similar to that seen in SCA1. Similar abnormalities, including decreased RORα expression and overlapping profiles of affected genes (*Itpr1*, *Calb1*, *Pcp4*, etc.), are observed in the cerebellum of transgenic mice with an SCA1 model [114]. CAG expansion in the ATXN1 gene enhances its interaction with Tip60, which disrupts RORα transactivation activity and leads to a decrease in the expression of genes necessary for the functioning and maintenance of Purkinje cell viability [115].

In mammalian cells, ATXN1–RBM17 and ATXN1–CIC complexes compete for binding to the common pool of ATXN1 protein. With the expansion of the polyglutamine tract in ATXN1, the proportion of ATXN1–RBM17 complexes increases, which corresponds to the mechanism of toxic gain-of-function [116,117]. This mechanism was confirmed in a Drosophila melanogaster model expressing ATXN1^[82Q]^, where increased formation of the ATXN1–RBM17 complex led to increased neurodegeneration of the eyes [118]. According to Tejwani et al. [119], specific abnormalities in the expression of CIC targets are observed in different types of cerebellar cells in SCA1. They are most pronounced in Purkinje cells, oligodendrocyte precursors, and microglia, manifesting as opposite changes: derepression (*Grid2*, *Cdh18*) and hyperrepression (*Nrxn1*, *Cntn4/5*, *Lrp1b*, *Nrg3*) of individual genes. In other populations of neural cells (unipolar brush cells, etc.), the profile of abnormalities differed, indicating the existence of additional mechanisms beyond CIC [120].

Normally, ATXN1 functions as a transcription regulator, affecting the expression of many genes, especially in cerebellar neurons. In mice lacking ATXN1, only mild neurobehavioral abnormalities are observed due to partial compensation by the ATXN1-like paralog (Boat1); however, the expression of many genes changes, especially those associated with Purkinje cell differentiation and synaptic plasticity [27]. Together with Boat1, it interacts with the RBP-J factor (CBF1), a key mediator of the Notch pathway, and in the absence of the Notch signal, it co-represses the target genes of this pathway, suppressing their transcription [86]. Thus, ATXN1 is integrated into the nuclear regulatory networks of neurons and influences transcription and, as has been shown, the maturation of Purkinje cells [121].

The central fragment of ATXN1 (amino acids 226–552) is necessary for binding to RNA and has splicing-modulating activity [92]. The C-terminal region (700–816 aa), which includes NLS and the AXH domain, is also required for the full realization of this function [122]. Previously, it was reported that RBM17 interacts with the protein through a linear motif that overlaps with NLS in the presence of phosphorylation at Ser776 [115]. In an experiment, Ohki et al. [122] showed in the HEK293 cell line that mutations eliminating the NLS region and the phosphorylated residue Ser776 do not disrupt the splicing activity of ATXN1. Even in the absence of these regions, the protein retained its ability to enhance exon inclusion in the minigene. This may indicate the presence of alternative splicing regulatory mechanisms unrelated to NLS and Ser776 [36]. In a recent study, Chen et al. [93] used ChIP-seq and RIP-seq methods to show that polyglutamine expansion has virtually no effect on the ability of ATXN1 to bind to RNA targets, but significantly affects its protein interactions and DNA-binding activity. Specifically, wild-type ATXN1 was shown to interact with the DNA replication/repair proteins MCM2, GNAS, and TMEM206, whereas mutant ATXN1 loses these interactions. Transcriptomic analysis of the brains of SCA1 mice revealed alternative splicing abnormalities involving at least 17 different splicing factors [121]. ATXN1 has the potential to interact with a wide range of splicing regulators, including RBFOX1, U2AF65, and RBPMS. Shorrock et al. [123] found that even before the onset of symptoms in SCA1 mice, there is massive splicing disruption (predominantly exon skipping) in the cerebellum, pons, and medulla oblongata. The absence of concomitant changes in mRNA levels during splicing disruption of key genes (*SLC35E2A*, *ZNF880*, *LRRC15*, *EXOSC10*) proves its role as an independent pathogenic mechanism [124]. In Drosophila models, Rbfox1 expression was critical for neuron survival in the presence of mutant ATXN1. A recent study on brain cell cultures [36] showed that excess *Rbfox1* enhances the death of neurons with ATXN1^[82Q]^, while suppression of *Rbfox1* protects cells from toxicity. Taken together, these data confirm the contribution of splicing program imbalance (via *RBM17*, *Rbfox1*, etc.) to degeneration in SCA1.

The normal ataxin-1 protein plays a critical role in the response to double-strand DNA breaks, effectively recruiting to sites of damage in the nucleus. The mutant form of the protein with polyglutamine expansion loses this function [125]. In Drosophila melanogaster models, expression of mutant ATXN1^[82Q]^ leads to hyperactivation of ATM kinase, and reducing its level significantly improves motor deficits, confirming the pathogenic role of DNA damage response impairment [125]. A similar mechanism of chronic activation of ATM/ATR signaling is observed in Huntington’s disease, indicating a common pathway of neurodegeneration for polyglutamine diseases [125,126]. This mechanism is confirmed by the detection of accumulated phosphorylated ATM substrates and the DNA damage marker γH2AX in patient cells, with this effect being potentiated by oxidative stress.

A systematic in vivo screen of all Drosophila DNA repair genes by Barclay et al. [127] revealed that ataxin-1 physically interacts with replication protein A (RPA), which mitigates neurodegeneration. RPA1 and its partners BRCA1/2 form a protective complex, and mutant ATXN1 disrupts the normal dynamics of RPA1 in the nucleus after DNA damage. Cell models have shown that increased expression of RPA (RPA1-3) inhibits repeat expansion, whereas an alternative form of RPA (involving the non-standard subunit RPA4, forming the so-called alt-RPA complex) stimulates the continuation of repeat expansion [128]. It has been shown that an imbalance in RPA complexes leads to competition between the standard and alternative RPA complexes (with RPA4), affecting the accuracy of repeat replication [128]. A recent study comparing the brains and blood of patients found that the level of repeat instability depends on the tissue: in the cerebellum, mosaicism is minimal with high expression of DNA repair genes, while in the cerebral cortex, maximum instability and pronounced mosaicism are observed against a background of low expression of repair proteins [129]. AAV-mediated delivery of RPA1 into the cerebellum improves motor function, normalizes DNA damage markers (γH2AX, 53BP1), and restores the morphology of Purkinje cells, with the effect persisting for almost a year after a single injection [130]. Mutant ATXN1, by binding to VCP/p97 through the polyQ domain, disrupts the dynamics of this key DNA repair factor [55].

It has been shown that ATXN1 directly binds to the transcription repressor GFI1. Knockout of the GFI1 gene (or its ortholog Senseless in Drosophila) completely eliminates the neurodegeneration caused by expression of mutant ATXN1, demonstrating the critical dependence of toxicity on this protein [111]. Mutant ATXN1 either hyperactivates the repressor complex with GFI1 or, by binding to it, disrupts its normal regulatory function [131].

**Figure 2:**
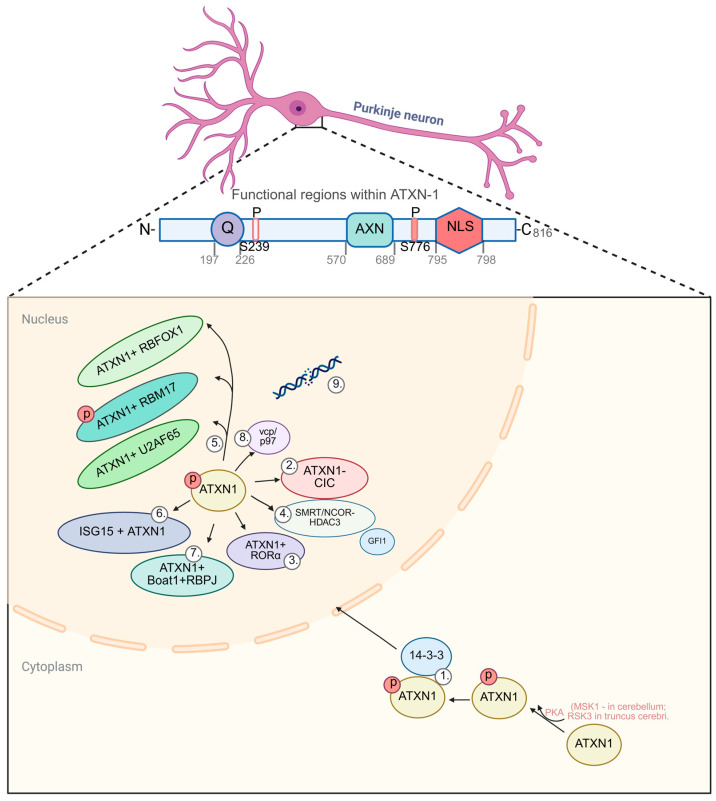
Pathogenesis of ATXN1 toxicity in SCA1. Schematic representation of ATXN1 and its key interactions in the cytoplasm and nucleus of Purkinje cells that determine protein toxicity in SCA1. Numbered arrows indicate the major molecular consequences of mutant ATXN1 activity: 1—Soluble cytoplasmic form; 14-3-3 protects against proteasome degradation; 2—Hyperrepression of a number of genes: *NRXN-1*, *Cntn4*, *cntn5*, *Lrp1b*, *Nrg3*, etc.; 3—Suppression of RORα-dependent transcription (*Itpr1*, *calb1*, *Pcp4*, etc.); 4—Enzymatic hypoacetylation of histones, reduction in transcription of target genes, enhancement of the repressive function of GFI1.; 5—Alternative splicing disruption: derepression (*GRID2*, *cdh18*), hyperrepression of certain genes (*Nrxn1*, *cntn4/5*, *Lrp1b*, *Nrg3*) in Purkinje cells, oligodendrocyte precursors, microglia; 6—ISG15, a protein structurally similar to ubiquitin, disrupts the degradation of a mutant protein, contributing to its pathological accumulation; 7—Interaction with the key mediator of the Notch pathway, CBF1(RBPJ); 8—Disruption of the dynamics of a key repair factor (VCP/p97). Additionally, ATXN1 disrupts the normal dynamics of RPA1 in the nucleus, which may stimulate further expansion of the repeats; 9—The mutant protein impairs double-strand break repair. Created in BioRender. Mayasin, Y. (2025) https://BioRender.com/v5wkved (accessed on 26 November 2025).

Another important mechanism is the recruitment of ATXN1 to co-repressor complexes such as SMRT/NCoR–HDAC3. This leads to enzymatic hypoacetylation of histones and sustained suppression of target gene transcription [86]. At the same time, HDAC3 activity itself is necessary for neuronal survival, and its mild inhibition can partially correct transcriptional defects in SCA1 [132]. Key steps in ATXN1-mediated pathogenesis in SCA1 are summarized in Figure 2.

## 4. Chapter 2. In Vitro Models

In vitro cell models are an indispensable tool for studying the molecular basis of SCA1. These systems allow for detailed investigation of the consequences of CAG repeat expansion in the ATXN1 gene, including protein conformational changes, alterations in protein–protein interactions, and dysregulation of cellular signaling pathways. Thanks to the possibility of genetic manipulation and high reproducibility of experiments, in vitro models serve as an optimal platform for primary screening of therapeutic compounds and evaluation of the effectiveness of gene-targeted approaches, such as ASOs, RNA interference, and CRISPR/Cas9 systems. In Table 2, we summarize the cell lines used to model SCA1, grouped by exogenous versus endogenous systems, indicating model type, expressed ATXN1 variant, and representative sources; practical notes are outlined below. This review systematizes data on the main types of cell lines used to model SCA1, providing researchers with a methodological basis for selecting the most relevant experimental system in accordance with specific research objectives.

### 4.1. Neuro-2a

Transient expression of human ATXN1^[82–85Q]^ in Neuro-2a cells recapitulates its predominantly nuclear localization and rapid formation of nuclear inclusions with liquid-phase condensation properties; acute stress induces enlargement and partial solidifi-cation of these condensates [90]. Phosphorylation at Ser776 and binding to 14-3-3 proteins modulate aggregate morphology and toxicity, whereas the S776A mutation reduces stress-induced changes [90,106]. Large-scale proteomic analysis identified ~675 ATXN1^[5Q]^ interaction partners, including key nucleocytoplasmic transport factors (im-portin-β1, NUP98, RanGAP1), which are sequestered by aggregates, disrupting nuclear-cytoplasmic transport; similar alterations were confirmed in Purkinje cells of ATXN1^[82Q]^ mice [88]. A shift in interactions is also characteristic: weakened ATXN1–CIC/U2AF65 and enhanced ATXN1–RBM17 binding, consistent with a toxic gain-of-function mechanism [116,117]. Additionally, interactome mapping revealed selective loss of protein–protein contacts while RNA-binding capacity was preserved [93]. The Neuro-2a model is widely used for primary screening: testing silencing approaches (ASO, siRNA, shRNA) and editing strategies (CRISPR/Cas9 targeting the CAG tract), demonstrating reduced ATXN1 levels and correction of cellular phenotypes [56]. It is also suitable for testing small molecules that modulate aggregation or degradation [132]. Key advantages include rapid experimental turnaround, reproducibility, and high transfection efficiency. However, it lacks Purkinje cell context and tissue organization, necessitating validation in primary neurons, iPSC-derived models, and in vivo systems.

Strengths: Rapid experimental turnaround, high transfection efficiency and good reproducibility, enabling efficient mechanistic and screening studies.

Pitfalls: Absence of Purkinje cell identity and cerebellar tissue architecture limits direct translational relevance and requires follow-up in more specialized neuronal and in vivo models.

Utility: Particularly useful for primary assessment of molecular interventions (ASO, siRNA, shRNA, CRISPR/Cas9) and small molecules targeting ATXN1 aggregation, stability, or interactions.

### 4.2. HEK-293

HEK293 cells are used for transient expression of both normal and mutant ataxin-1, providing a platform for microscopy and biochemistry. Stable HEK-293T lines express human ATXN1 with tandem tags (FLAG, SBP) [93], GFP-ATXN1[82Q] [37], and are employed for co-expression of signaling regulators (e.g., FLAG-CBF1, FLAG-Su(H), FLAG-HES1) [133]. The role of S776 phosphorylation in inclusion formation has been studied in this line. The model is suitable for immunoprecipitation and tandem affinity purification of interactomes; studies in HEK-293T showed selective loss of protein–protein (MCM2, GNAS, TMEM206) and protein-DNA interactions while RNA-binding capacity was retained [93]. RNA interference (shRNA and artificial miRNA) is effective [134,135,136], and pathways such as Notch [133] and ERK [137], alternative splicing [122], and inducible ubiquitin-dependent degradation of ATXN1 [138] have been investigated. This model offers simplicity, high transfection efficiency, reproducibility, and suitability for co-IP, ChIP-seq, RIP-seq, and primary chemogenetic screening. However, its non-neuronal origin and lack of cerebellar context limit interpretability, requiring validation in neuronal models and in vivo systems.

Strengths: Simple, robust system with high transfection efficiency, ideally suited for biochemistry, interactome mapping and various -seq based assays.

Pitfalls: Non-neuronal kidney origin and absence of cerebellar context restrict direct extrapolation to neuronal pathophysiology.

Utility: Well-suited for dissecting ATXN1 post-translational regulation, interaction networks, signaling pathways and for primary chemogenetic or genetic modifier screens prior to validation in neuronal models.

### 4.3. HeLa

HeLa cells, similar to HEK293 cells, are used for transient expression of both normal (33Q) and mutant ATXN1[86Q]. Characteristic SCA1-associated punctate intranuclear inclusions form in HeLa cells, and their dynamics are recorded [139,140]. The model is used to study the co-sequestration of partners in inclusions (e.g., VCP/p97 and DNA repair factors) [139] and to analyze signaling pathways, including Wnt/β-catenin [46]. HeLa cells are actively used in miRNA experiments, demonstrating a decrease in ATXN1 levels [139], as well as for primary screening of silencing (ASO, siRNA, shRNA) and editing (CRISPR/Cas9 on the CAG region) with ATXN1 reduction and phenotype correction [56,136]. This model provides extremely high transfection efficiency and clear nuclear morphology, making it convenient for immunofluorescent visualization of ATXN1 inclusions and basic biochemical measurements. At the same time, the tumorigenic, non-neuronal origin and lack of cerebellar context limit the transferability of conclusions; key findings should be confirmed in neuronal systems and in vivo models.

Strengths: Extremely high transfection efficiency and clear nuclear morphology, allowing precise visualization and quantification of ATXN1 inclusions and associated factors.

Pitfalls: Tumorigenic, non-neuronal origin without cerebellar specificity reduces physiological relevance and mandates confirmation in neuronal systems.

Utility: Appropriate for imaging-based studies of inclusion dynamics, co-sequestration of partners, pathway interrogation and primary screening of silencing/editing strategies (ASO, siRNA, shRNA, CRISPR/Cas9).

### 4.4. DAOY

In the DAOY cell line (human cerebellar medulloblastoma), the formation of aggregates is reproduced, and the nuclear-cytoplasmic distribution of mutant ATXN1 is studied [90], as well as the intercellular spread of ATXN1 inclusions [97]. In DAOY, it has been shown that inhibition of PKA phosphorylation of ATXN1 accelerates the degradation of the mutant protein [41]. High-throughput screening revealed that suppression of the RAS–MAPK–MSK1 cascade reduces mutant ATXN1 levels [75], with PAK1 identified as a modulator of its levels [141,142]. This model is widely used for shRNA- and siRNA-mediated silencing [75,84,108]. The medulloblastoma line provides a relevant cerebellar cell background, supports stable expression of ATXN1 constructs, and scales well for combined chemical-genetic screens. However, its tumorigenic nature and high proliferation may distort neuron-specific effects, so critical observations require validation in iPSC-derived neurons and in vivo systems.

Strengths: Cerebellar-derived background, stable ATXN1 expression and scalability make it suitable for medium-to-high-throughput chemical and genetic screens.

Pitfalls: Tumorigenic origin and high proliferation rate can confound neuron-specific biology and long-term differentiation-related phenomena.

Utility: Particularly useful for identifying pathway-level modulators of ATXN1 levels or stability (e.g., RAS–MAPK–MSK1, PAK1) and for RNAi-based silencing screens before transfer to iPSC-derived neurons and in vivo models.

### 4.5. MSC

A stable human mesenchymal stem cell line with an integrated Tet-On YFP-ATXN1^[Q82]^ construct accumulates insoluble nuclear aggregates within 10 days; this inducible model allows detection of specific molecular changes in vitro, potentially relevant to neuropathology development in vivo [49]. Inducible expression enables precise control of gene activation and aggregate accumulation kinetics, facilitating multi-timepoint “omics” analyses and testing interventions at different stages. As these are dividing non-neuronal cells, mechanistic conclusions require confirmation after differentiation and/or in co-cultures with neurons, followed by in vivo validation [96].

Strengths: Inducible system with tight temporal control over ATXN1 expression, well-suited for studying aggregation kinetics and stage-specific molecular changes.

Pitfalls: Non-neuronal, proliferative nature limits direct relevance to post-mitotic cerebellar neurons and necessitates complementary neuronal models.

Utility: Appropriate for multi-timepoint omics, early mechanistic dissection and testing of interventions at defined stages of aggregate formation prior to validation in differentiated neuronal contexts.

### 4.6. SCA1 Patient Fibroblasts

Fibroblasts from SCA1 patient biopsies carry the endogenous mutant ATXN1 allele with expanded CAG repeats. Cultures exhibit energy metabolism alterations (reduced O_2_ consumption and mitochondrial activity), indicating respiratory chain dysfunction and oxidative stress [143]. Unlike neuronal models, large nuclear ATXN1 inclusions are not formed, but sensitivity to mutant protein accumulation via impaired cellular functions (apoptotic priming or transcriptional changes) is observed. Fibroblasts are actively used for testing gene and antisense approaches: allele-specific oligonucleotides (CUG)7 reduce mutant ATXN1 levels [144]; CRISPR/Cas9 with sgRNA pairs (G3N/G8N) induces large deletions in exons 8–9 and reduces mutant protein expression [145]; siRNA/ASO significantly lower ATXN1 transcript levels but act on both alleles [146]. Cultures retain the true genotype and show genetic stability with good transfection tolerance, making them suitable for primary assessment of molecular interventions. The limitation remains their non-neuronal specificity; thus, effects must be replicated in iPSC-neurons or other neuronal models and confirmed in vivo.

Strengths: Endogenous mutant ATXN1 context with preserved patient genotype and good transfection tolerance, enabling robust molecular manipulations.

Pitfalls: Non-neuronal peripheral cell type lacking cerebellar specialization limits direct mechanistic conclusions about neuronal pathogenesis.

Utility: Well-suited for primary assessment of molecular interventions (allele-specific oligonucleotides, ASO, siRNA, CRISPR/Cas9 strategies) and for detecting systemic cellular stress and metabolic alterations prior to studies in neuronal models.

### 4.7. SCA1 Patient iPSCs

iPSC technology enables reprogramming of SCA1 patient cells (fibroblasts/lymphocytes) to pluripotency and differentiation into “pan-neurons” or specific subtypes, including Purkinje cells. Such cultures exhibit nuclear and cytoplasmic ataxin-1 aggregates, axonal/dendritic shortening, reduced branching complexity, and delayed network maturation by MEA [143]. In Pappadà et al. [147], SCA1 fibroblasts were repro-grammed with non-integrating Sendai vectors (KOS, Klf4, c-Myc) into UNIFEi001-A iPSCs, forming compact round colonies with defined borders and high nucleus/cytoplasm ratios. iPSC-Purkinje cells show aberrant ISG15 activation, suppression of which improves differentiation [43]. Gene therapy approaches are actively tested: delivery of Cas9 and sgRNA pairs flanking the polyQ repeat in exon 8 of ATXN1 induces excision of the expanded region and reduces mutant protein levels by ~50% [136]; neuronal networks are suitable for small molecule screening [148]. Patient-specific iPSCs replicate the genomic background and allow differentiation into target types, including cerebellar neurons, modeling disease dynamics in human material. The cost of this relevance is high labor and expense, heterogeneity of differentiated cultures, and limited reconstruction of intercellular interactions, requiring rigorous experimental design and independent verification.

Strengths: Patient-specific human genomic background with the possibility to generate relevant cerebellar neuron subtypes, including Purkinje cells, and to model disease-related network phenotypes.

Pitfalls: Labor-intensive and costly workflows, culture heterogeneity and incomplete recapitulation of in vivo circuitry complicate standardization and interpretation.

Utility: Particularly valuable for mechanistic studies of SCA1 in human neurons, for testing gene-editing and gene-silencing strategies, and for secondary/tertiary small-molecule screening with higher translational relevance.

## 5. Chapter 3: In Vivo Models

For a systematic evaluation of experimental models used in the study of SCA1 pathogenesis, we conducted a quantitative analysis of original scientific publications indexed in the PubMed database. To minimize bias and ensure comparability of results, strict inclusion criteria were applied. The analysis included only works categorized as Classical Article, Clinical Trial, Randomized Controlled Trial, Veterinary (animal experiments), as well as publications indicating various forms of support (Research Support), including N.I.H. Extramural/Intramural, Non-U.S. Gov’t, U.S. Gov’t, U.S. Gov’t P.H.S., and U.S. Gov’t Non-P.H.S. This selection allowed for a descriptive quantitative analysis of the frequency of use of various in vivo SCA1 models in peer-reviewed research. The exclusion of reviews and secondary sources ensured the formation of a corpus consisting exclusively of original studies, which is critically important for identifying the most representative experimental systems in this field.

An important part of the analysis was the comparison of phenotypic manifestations of SCA1 in experimental models. For this purpose, a three-level staging system (early, middle, and late stages) was used, based on a comprehensive assessment of neurological and cognitive impairments:

- Early stage: Minimal motor disorders, such as mild gait instability, usually in the absence of pronounced histopathological changes; cognitive functions are largely preserved, with only subtle deviations recorded.
- Middle stage: Pronounced neurological deficits, including ataxia and impaired motor coordination, are detected in behavioral tests; cognitive disorders (e.g., impaired learning or memory) become more distinct; at the cellular level, neuronal dysfunction, particularly in Purkinje cells, is observed, along with early signs of neurodegeneration.
- Late stage: Full-blown clinical picture with severe ataxia, tremor, and potential complications (e.g., dysphagia); cognitive impairments become pronounced and include disorientation and significant memory decline.

Additionally, a quantitative determination of the number of studies utilizing each model was conducted as of 2025, taking into account the inclusion criteria described above (Table 3). To facilitate this model selection process, we have developed a practical decision matrix (Table 4) that aligns key research objectives with the most suitable experimental models, based on our systematic analysis.

### 5.1. The ATXN1 Model ^[154Q/2Q]^ Knock-in

The most frequently cited in vivo model of SCA1 is the ATXN1^[154Q]^ knock-in line [149], with 31 citations as of 2025. In this model, one ATXN1 allele encodes a protein with a polyQ repeat of 154 amino acid residues. Expression of mutant ataxin-1 is driven by the endogenous promoter in all tissues, including the brain, spinal cord, and peripheral organs.

Impaired motor learning on the rotarod is detected from week 5 [62]. Cognitive deficits—specifically, spatial memory impairment in the Barnes maze—emerge by week 8 [77]. By week 12, more pronounced defects in spatial learning (Morris water maze) and fear-conditioned memory are observed, accompanied by astrocyte activation (GFAP positivity) and microgliosis in the cerebellum [150]. Behavioral studies demonstrate increased thigmotaxis in ATXN1^[154Q/2Q]^ animals compared to controls, reflecting anxiety-like behavior that progresses from weeks 6 to 26. During this period, acoustic startle response (ASR) and prepulse inhibition tests reveal heightened startle sensitivity, while the forced swim test (FST) indicates depression-like behavior, most pronounced between weeks 9 and 13 [73]. In the hidden platform test, ATXN1^[154Q/2Q]^ mice (9–12 weeks) show fewer target zone crossings than wild-type mice, indicating spatial memory deficits [132]. By week 30, pronounced kyphosis and hindlimb atrophy are evident; premature mortality occurs by week 32 [41] and further by weeks 35–45 [149]. During this period, motor and psychiatric phenotypes (anxiety, depression-like behavior) worsen sharply, correlating with Purkinje cell degeneration [46] and mitochondrial dysfunction in the hippocampus [73]. Mechanistic studies reveal that hyperactivity of molecular layer interneurons (MLINs) disrupts calcium signaling in Purkinje cell dendrites, driving motor deficits [151]. Dendritic atrophy of Purkinje cells primarily affects posterior lobes VII and X of the vermis and Crus I of the cerebellar hemispheres [152]. Inferior olivary neurons exhibit a distinct pattern: early dendritic hypertrophy followed by late somatic hypertrophy, accompanied by loss of calbindin, similar to Purkinje cells [153]. Reduced calbindin expression in Purkinje cells and cerebellar glomeruli positively correlates with neurodegeneration in SCA1 [132].

A notable feature is the respiratory phenotype. By week 33, heterozygous ATXN1^154Q^ animals develop progressive respiratory failure, including rapid shallow breathing, diaphragmatic weakness, and neurogenic changes in spinal motor neurons [154]. These manifestations are phenotypically akin to bulbar symptoms in patients and indicate degeneration of respiratory motor neurons, previously identified as a key factor in the premature mortality of SCA1 animals.

### 5.2. The ATXN1 Model^[78Q/2Q]^ Knock-in

The first knock-in line in which a mutant polyglutamine tract (78 CAG repeats) was inserted into the murine ATXN1 gene. This model replicates the endogenous expression level and natural tissue distribution of ataxin-1, mirroring the pattern observed in patients.

Heterozygous mice ^[78Q/2Q]^ exhibited repeat instability upon inheritance (minor shifts of ±2–6 repeats, particularly through maternal transmission), yet no ataxic phenotype was observed even in aged animals. Only homozygous 78Q/78Q animals showed moderate rotarod performance decline by 9 months, which was not accompanied by overt ataxia or histopathology [155]. Walking speed in the Barnes maze did not differ between ATXN1^[78Q/2Q]^ mice and their wild-type littermates [77]. Even at 18 months, these mice displayed no Purkinje cell loss or other neurodegenerative changes characteristic of transgenic models with strong overexpression of the mutant protein. These findings indicate that with moderately expanded polyQ (78Q) and physiological expression levels of ataxin-1, an extended duration of exposure is required to induce noticeable neurological deficits. Consequently, longer repeats were necessary to robustly model SCA1 in mice.

### 5.3. ATXN1^[82Q]^

The B05 line expresses human ATXN1 with an 82-CAG expansion under the Purkinje cell–specific Pcp2 (L7) promoter [156]. Owing to this tissue-restricted driver, pathology is largely confined to the cerebellum and does not recapitulate extra-cerebellar manifestations (e.g., bulbar dysfunction, cognitive deficits), which constrains the translatability of interventions targeting only this cell type [134]. Transgene expression is elevated ~50–100-fold relative to endogenous levels [156]. Mutant ataxin-1 accumulates in Purkinje cell nuclei, forming intranuclear inclusions and producing progressive cerebellar ataxia. The phenotype emerges early: by ~5 weeks, mice exhibit impaired performance on the accelerating rotarod; by 12 weeks, a mild coordination deficit becomes evident as head bobbing during ambulation [33]. By 6 months, gait is overtly ataxic, and animals frequently lose balance when rearing on their hind limbs. Histopathology evolves from 3–4 weeks with vacuolation of Purkinje cell somata; between 6–12 weeks, there is a marked reduction in calbindin (CaB) and parvalbumin (PV) in Purkinje cells [157]. By ~12 weeks, pronounced defects in mGluR1-dependent signaling and reduced synaptic plasticity in the molecular layer accompany frank ataxia and motor incoordination; Purkinje cell loss is still limited at this stage [59]. Subsequently, dendritic atrophy is detectable by ~15 weeks, with ~32% Purkinje cell loss by ~24 weeks, and by ~27 weeks, dendritic arbors are markedly shortened and flattened with a substantial reduction in surviving neurons [33]. Notably, degeneration is mosaic: the flocculonodular lobe is relatively spared compared with anterior regions [51]. The late stage (~52 weeks) provides a window to interrogate alternative-splicing dynamics across disease progression while >50% of Purkinje neurons remain, facilitating stage-resolved analyses [36]. AAV-delivered artificial microRNA targeting human ATXN1 (rAAV.miS1) administered to symptomatic B05 mice partially improved motor performance and neuropathology [134].

In transgenic mice, expression of the non-phosphorylatable ATXN1^[82Q]-S776A^ variant prevents neurodegeneration, likely by promoting protein turnover and weakening the interaction with the splicing factor RBM17, whereas the phosphomimetic substitution with aspartate strengthens this interaction and exacerbates toxicity [50]. As demonstrated by Fagan et al. [136], CRISPR/Cas9-mediated reduction in mutant ataxin-1 by ~20% improves motor performance in B05 mice; moreover, combining RNAi/ASO with targeting of the ATXN1L paralog yields an additive therapeutic effect despite the presence of ~25–30 genomically integrated cDNA copies of ATXN1 in this model.

Using the ATXN1^[82Q]^ mouse model, Kim et al. [158] showed that inhibiting NF-κB signaling in astrocytes before the onset of motor deficits aggravates disease progression, whereas late-stage inhibition ameliorates motor function, indicating a stage-dependent—and potentially deleterious—role of astrocytes at advanced stages of SCA1.

### 5.4. ATXN1^[30Q]D776^

The ATXN1^[30Q]-D776^ model was generated to interrogate the role of serine-776 phosphorylation in SCA1 pathogenesis. It expresses human ATXN1 with a normal polyQ length (30 CAG) but carries a serine-to-aspartate substitution at position 776 (S776D) to mimic constitutive phosphorylation. Transgene expression is restricted to Purkinje cells by the Pcp2 (L7) promoter, and the line was maintained on an FVB/N background [159]. Despite the absence of an expanded polyQ tract, ATXN1^[30Q]-D776^ mice exhibit morphological abnormalities, including defects in Purkinje cell interactions with granule cell precursors—specifically, reduced translocation of precursors along Purkinje dendrites and diminished detachment of their endfeet from the Purkinje soma [53]. Notably, progressive age-dependent neurodegeneration typical of classical SCA1 models does not develop in these animals [159].

In the cerebellum of ATXN1^[30Q]-D776^ mice, the peptide hormone cholecystokinin (CCK) is upregulated [50]. It has been proposed that CCK overexpression and activation of the CCK1 receptor (CCK1R) exert a compensatory effect that prevents progressive Purkinje cell degeneration; consistent with this, genetic ablation of CCK1R in this background precipitates a fully penetrant degenerative phenotype with Purkinje cell loss [110]. Pharmacological activation of CCK1R normalizes dysregulated mTORC1 signaling, mitigates Purkinje cell injury, and improves motor performance [50,160]. Collectively, these data underscore a functional interplay between ATXN1 S776 phosphorylation and CCK signaling, highlighting CCK1R as a potential therapeutic target in SCA1.

### 5.5. ATXN1^[82Q]D776^

Using the B05 transgenic line, the role of phosphorylation at serine 776 in ataxin-1 was elucidated. This site governs the stability and protein–protein interactions of the mutant protein. Substitution of Ser776 with a non-phosphorylatable alanine (S776A) markedly attenuates the toxicity of the long-polyQ variant: mice carrying ATXN1^[82Q]-S776A^ do not develop overt ataxia at the same repeat length, exhibit only mild Purkinje cell abnormalities, and show minimal motor impairment (no frank ataxic gait and only subtle deficits on the accelerating rotarod) [106].

### 5.6. f-ATXN1^[146Q/2Q]^

The f-ATXN1^[146Q/2Q]^ model is a conditional allele in which site-specific recombination between FRT and LoxN sites mediates replacement of the coding sequence of one murine ATXN1 allele with human ATXN1 harboring a pathogenic CAG expansion (146 repeats), while the second allele remains wild type (2Q). This strategy contrasts with the KI-154Q full knock-in, in which both ATXN1 alleles are permanently replaced by human ATXN1 with 154 CAG repeats in all cells. A key advantage of the conditional design is spatiotemporal control of mutant protein expression via tissue-specific induction of Cre/Flp recombinases. For example, Duvick et al. [13] crossed f-ATXN1 mice to ACTA1-Cre, affecting muscle-restricted inactivation of mutant ATXN1 and preventing myopathy: offspring of f-ATXN1; ACTA1-Cre lacked muscle fiber atrophy and retained normal forelimb grip strength, whereas littermates with active mutant ATXN1 exhibited marked calf muscle wasting and weakness. Eliminating mutant ATXN1 in all neurons with Nestin-Cre substantially delayed ataxia onset and extended lifespan, indicating the central nervous system as the primary driver of disease, while striatal-restricted deletion using Rgs9-Cre had minimal impact, suggesting a lesser contribution of striatal neurons at early stages [13].

Behaviorally, f-ATXN1^[146Q/2Q]^ mice show a significant deficit on the accelerating rotarod by 6 weeks that worsens with age. By 12 weeks, an open-field assay reveals reduced distance traveled versus controls; at 24 weeks, impairments emerge on the Barnes maze, and by 36 weeks, animals exhibit a hindlimb clasping phenotype. Concomitantly, histological and functional assessments demonstrate atrophy of hindlimb muscles—including tibialis anterior and extensor digitorum longus—and reduced grip strength compared with controls. Notably, Selimovic et al. [80] reported pronounced sex differences: by 12 weeks, males display more severe motor impairment (rotarod and balance beam) and reduced contextual fear freezing, whereas cognitive deficits in females (Barnes maze) become evident only by 24 weeks; moreover, males begin to lose body mass by 33–35 weeks, while female body weight remains stable [80]. Collectively, these findings underscore the multisystem impact of mutant ATXN1 and highlight the importance of sex as a biological variable in SCA1 pathogenesis.

### 5.7. Drosophila Melanogaster ATXN1^[82Q]^

In the Drosophila melanogaster model, the complete coding sequence of human ATXN1 with an expanded polyQ tract (82 CAG) is expressed using the GAL4/UAS yeast hybrid system.

Expression of ATXN1[82Q] in the photoreceptors of the fly eye leads to the formation of nuclear protein inclusions in retinal cells and CNS neurons, causing photoreceptor degeneration and neurodegenerative changes [118]. Further studies in Drosophila revealed key molecular mechanisms of SCA1: Tsuda et al. [111], found that the conserved AXH domain of Ataxin-1 interacts with the transcription factors Senseless (Drosophila) and Gfi-1 (mammalian), with excess Ataxin-1 suppressing Senseless, causing sensory organ development defects, and a mutation in the AXH domain blocking this effect. Mizutani et al. [161] discovered a paralog of ATXN-1–ATXN1-Like, which attenuates the toxicity of mutant Ataxin-1 in Drosophila by suppressing ATXN182Q-induced eye defects, and also showed that ATXN1L levels are reduced in affected neurons in a mouse model of SCA1. Interestingly, elevated levels of ANTN1L displace mutant ATXN1 from its natural complex with CIC, which suppresses selective neuropathological phenotypes [162]. Ramahi et al. [83] demonstrated that the related protein Ataxin-2 (dAtx2) strongly influences the toxicity of Ataxin-1^[82Q]^ in Drosophila: increasing its level enhances damage, while decreasing it attenuates degeneration, with mutant Ataxin-1 causing accumulation of dAtx2 in nuclei. Tong et al. [133] linked SCA1 to the regulation of Notch signaling, showing that Ataxin-1 and its relative BOAT1 are involved in the Notch pathway, where BOAT1 reduces its activity by interacting with components of the signaling complex. Park et al. [75] revealed that the RAS–MAPK–MSK1 signaling cascade controls Ataxin-1 levels, and that reducing its activity decreases ATXN1 stability and attenuates neurodegeneration in an SCA1 model, suggesting a new therapeutic target. In recent studies, Palmer et al. [163] showed that motor dysfunction is associated with dysregulation of matrix metalloproteinases, which affect extracellular matrix signaling factors, as well as changes in SMN levels at the neuromuscular junction. Such transgenic flies showed age-related deterioration in motor function: males lived shorter than control individuals and demonstrated a progressive decline in overall motor activity and vertical movement ability (geotaxis). The study by Hunter et al. [164] also observed pronounced sexual dimorphism not only in motor function, but also in the expression of circadian genes, markers of oxidative stress, and the level of expression of glial phagocyte receptors. A limitation of the Drosophila model is the absence of a cerebellum in the insect, which prevents direct investigation of the specific degeneration of Purkinje cells characteristic of SCA1. Nevertheless, Drosophila is indispensable for rapid analysis of molecular mechanisms and primary testing of therapeutic approaches for polyQ diseases.

### 5.8. Danio Rerio^[82Q]^

The model expresses human mutant ATXN1 with 82 CAG repeats, with the coding sequence derived from an SCA1 patient harboring a continuous tract of 82 glutamines [165]. Purkinje cell degeneration is regionally patterned, being markedly more pronounced in rostral cerebellar domains than in caudal regions, recapitulating the “mosaic” pattern reported in B05 transgenic mice. Despite substantial Purkinje neuron loss, adult fish exhibit no gross deficits in basic swimming performance; however, reduced exploratory behavior in novel environments is detected in the context of pronounced neuronal loss. The system enables real-time in vivo visualization of Purkinje neurodegeneration and supports rapid screening of neuroprotective candidates, serving as a complementary platform to mammalian studies [165].

A genome-wide analysis in Danio rerio identified two human ATXN1 orthologs—ATXN1a and ATXN1b—and the related ATXN1l locus; at transcript and protein levels, all three genes are expressed in the developing cerebellum and in mature Purkinje cells, with ATXN1b initiating expression at early brain patterning and becoming predominantly localized to the cerebellum during development. These data underscore the conservation of ATXN1 function across vertebrates and support the suitability of zebrafish for modeling SCA1 pathogenesis [166]. In a transgenic line with Purkinje cell–restricted expression of human ATXN1^[82Q]–GFP^, investigators observed progressive Purkinje cell loss and a mosaic cerebellar involvement [165].

### 5.9. Nonhuman Primate Models

Generating stable SCA1 transgenic models in nonhuman primates remains extremely challenging. Unlike Huntington’s disease (for which transgenic macaques have been produced), attempts to create a germline SCA1 mutant in primates are hampered by practical and ethical constraints. Primates are costly to house, have slow reproduction rates, and require specialized husbandry and oversight [167]. These factors make breeding and selecting for a disease transgene very difficult. Consequently, researchers have mainly used primates for therapeutic testing rather than for producing an endogenous disease phenotype.

Specifically, adeno-associated viral vectors encoding microRNA designed to silence ATXN1 were injected into the cerebellum of adult rhesus macaques, achieving efficient transduction of Purkinje cells and other cerebellar neurons and producing >30% reduction in endogenous ataxin-1 without overt adverse events during the initial post-delivery period [168]. Importantly, however, extended observation (~3 months) revealed focal cerebellar neurotoxicity attributable to nonspecific activity of the viral promoter, underscoring a key translational constraint and the need for promoter/restrictor strategies that ensure cell-type specificity and safety in large brains [168].

### 5.10. LPS Model

To interrogate the contribution of neuroinflammation to SCA1, bacterial endotoxin lipopolysaccharide (LPS)—a potent inducer of innate immune signaling—is employed. Acute LPS injections in healthy rodents elicit robust glial activation in the brain. Systemic LPS administration selectively activates the transcription factor c-Jun in Bergmann glia of the cerebellum, evidenced by nuclear c-Jun phosphorylation in S100β-positive astrocytes of the Purkinje cell layer, mirroring patterns observed in cerebella from deceased SCA1 patients [169]. Notably, astrocyte-restricted inhibition of NF-κB signaling in SCA1 mice worsened disease manifestations at early stages, whereas inhibition at late stages improved motor performance, underscoring stage-dependent, bidirectional roles of astroglial NF-κB in SCA1 [150].

### 5.11. Ara-C Model

Systemic cytarabine (Ara-C) exposure in neonatal mice penetrates the brain and selectively ablates proliferating cerebellar granule neurons, leading to underdevelopment of the granule layer and secondary degeneration of Purkinje cells. In experimental settings, daily Ara-C during the first three postnatal days produced a persistent ataxic phenotype: by postnatal week 10, mice exhibited ~65% reduction in rotarod retention relative to controls [170]. Morphological analyses revealed marked cerebellar volume loss, decreased neuronal counts (NeuN expression) and a pronounced reduction in calbindin in surviving Purkinje cells.

### 5.12. Ethanol Model

Chronic ethanol exposure in rodents serves as a non-genetic ataxia model that recapitulates the selective vulnerability of Purkinje cells. Long-term liquid-diet ethanol (~35% of calories from ethanol for 8–10 months) drives reduction in Purkinje dendritic arborization and loss of dendritic spines [171]. Electron microscopy demonstrates dilation of smooth endoplasmic reticulum, vacuolization, and formation of degenerative bodies within dendrites, consistent with chronic cellular stress and neuronal remodeling [172].

Functionally, animals display impaired motor coordination, reduced speed and movement accuracy, and generalized hypolocomotion [173,174], with the most pronounced phenotypes in aged cohorts, indicating age-dependent loss of compensatory capacity. Although devoid of a genetic lesion, ethanol-induced ataxia provides a tractable platform to screen neuroprotective and symptomatic interventions aimed at preserving cerebellar function—e.g., agents stabilizing calcium homeostasis, limiting oxidative stress, and supporting synaptic plasticity [175,176].

**Table 3:** In vivo models for SCA1.

Model	Modification	Clinical Features	Cellular and Molecular Changes	Number of Papers that Employ This Model *	Source	Supplier
ATXN1^[82Q]^, mouse	Expresses the full-length human SCA1 cDNA containing 82 uninterrupted CAG repeats under the control of the Purkinje cell-specific Pcp2 promoter.	Early stage: At week 5, impaired performance on the rotarod. By week 12: mild coordination impairment, evident as “head bobbing during walking” [33]	At 6–12 weeks, transgenic mice exhibit a severe reduction in CaB and PV in Purkinje cells [59]. By week 12, mild Purkinje cell loss, reduced synaptic plasticity in the cerebellar molecular layer, and impaired mGluR1 signaling are observed [59]. At week 15, Purkinje cell dendritic atrophy is evident, progressing to approximately 32% Purkinje cell loss by week 24. By week 27, Purkinje cell dendrites are severely shortened and flattened. At 35 days, Purkinje cell dysfunction is localized to the anterior cerebellar region. By 1 year, significant Purkinje cell death occurs.	28	Burright et al. [26]	The B05 strain is not officially listed in any major repository (e.g., IMSR, Jackson Lab, EMMA). The Tg(Pcp2-ATXN1*82Q) line is likely available only through direct contact with the Harry T. Orr laboratory.
Knock-in ATXN1^[78Q/2Q]^, mouse	Targeted 78 CAG repeats into the endogenous mouse locus. Thus, one ATXN1 allele in these mice contains 78 glutamines, the second remains normal (2Q).	Early stage: No pathological changes. Middle stage (9 months): Significant rotarod deficits [155]. Late stage: No visible ataxia up to 18 months [155]. The 78Q copy number is insufficient for complete disease manifestation within the standard mouse lifespan [70].	Not observed	3	Lorenzetti et al. [155]	The line is available exclusively through direct contact with the Harry T. Orr laboratory or corresponding authors of the original publications.
Knock-in ATXN1^[154Q/2Q]^, mouse	Targeted 154 CAG repeats into the endogenous mouse locus. Thus, one ATXN1 allele in these mice contains 154 glutamines, the second remains normal (2Q).	Early stage: Impaired motor learning on rotarod from week 5 [62]. Middle stage: Spatial memory deficits (Barnes maze) from week 8 [77]; increased anxiety (thigmotaxis in open field), enhanced acoustic startle response (ASR) and prepulse inhibition deficits from weeks 6–26; depression-like behavior (forced swim test) from weeks 9–13 [73]. Late stage: Pronounced kyphosis and hindlimb atrophy from week 30; premature mortality observed from week 32 [69] to weeks 35–45 [149].	Week 3: Purkinje cell count unchanged; reduced molecular layer thickness [57]. From week 4: Impaired dendritic arborization [67]; progressive postsynaptic destabilization and reduced synaptic scaffolding protein expression. From day 40: Decreased Homer-3 levels [134]; activation of IFNβ/STAT1 pathway (ISG15 cytokine). Week 8: Significant astrogliosis (GFAP ↑), Bergmann glia hypertrophy, and microgliosis [35]. Week 12: ATXN1 nuclear aggregates in motor neurons. Week 24: ATXN1 nuclear aggregates in Purkinje neurons [154]. Metabolic changes: Elevated glutamine and total creatine levels vs. wild-type [136].	31	Watase et al. [149]	Commercially available at Jackson Laboratory (Stock No. 005601).
f-ATXN1^[146Q/2Q]^, mouse	Mouse f-ATXN1^[146Q/2Q]^ with mouse ATXN1 coding exons replaced by human ATXN1 exons encoding 146 glutamines	Early stage: Rotarod deficit at 6 weeks [13]; impaired locomotor function (open field test) at 12 weeks. Middle stage: Kyphosis onset at 12 weeks; cognitive deficits (Barnes maze) at 24 weeks. Late stage: hindlimb clasping phenotype and markedly reduced lifespan at 36 weeks.	Nuclear accumulation of mutant ATXN1 and disruption of key intracellular signaling pathways, including impaired ATXN1 phosphorylation.	2	Duvick et al. [13]	The mouse model is registered at Jackson Laboratory.
ATXN1^[154Q_flox_stop/+]^, mouse	A loxP-flanked stop cassette was inserted via CRISPR/Cas9 into the intron upstream of the first coding exon of mutant ATXN1[154Q].	Early stage (7–15 weeks): Reduced distance traveled in open field test. Middle stage (28 weeks): Significant weight loss. Late stage (72 weeks): Respiratory insufficiency; markedly reduced lifespan by 75 weeks [177].	From 3 weeks: Two-fold increase in ATXN1 mRNA levels, while producing less than half of the pathogenic protein compared to the ATXN1^[154Q]^ [177].	0	Orengo et al. [177]	This conditional line is likely available only upon request from the study authors or through collaboration with their laboratory at Baylor College of Medicine.
ATXN1^[30Q]D776^, mouse	Mouse expresses the complete human ATXN1 gene with normal polyQ length (30 glutamines) but with a serine to aspartate substitution at position 776 (S776D)	Early stage: Impaired development of spinocerebellar connections: reduced climbing fiber translocation along Purkinje cell dendrites and poor synaptic wiring [53]. Middle stage: Mild neurological deficits [13]. Late stage: No pronounced pathology observed.	Retain synaptic impairments but do not develop progressive neuronal loss [13].	3	Duvick et al. [13]	This line is likely available only upon request from the study authors
ATXN1^[82Q]^, Drosophila melanogaster	Human ATXN1^[82Q]^ protein expression is driven by the UAS promoter using the yeast UAS/GAL4 hybrid system.	Middle stage: Retinal degeneration and reduced visual function in flies. Late stage: Complete retinal degeneration and vision loss 122].	Formation of nuclear inclusions in retinal photoreceptors and CNS neurons, retinal degeneration	4	Fernandez-Funez et al. [118]	Available from the Bloomington Drosophila Stock Center; stock number P{UAS-ATXN1.82Q}F7 (BDSC #8146).
ATXN1^[82Q]^, Danio rerio	Expression of human protein ATXN1^[82Q]^. The construct includes Purkinje-specific regulatory elements (8×cpce under the E1b basal promoter) and the membrane-targeted red fluorophore GAP-mScarlet.	Early stage: After 1–2 months: decrease in exploratory behavior in the “new aquarium.” Middle stage: decrease in swimming and coordination. Late stage: pronounced disturbances in swimming and balance [178].	Middle stage: progressive, age-dependent degeneration of Purkinje cells in the cerebellum. Late stage: massive death of Purkinje neurons.	0	Elsaey et al. [165]	The authors note that the transgenic fish described in the paper are available “upon request.”
Ara-C, mouse	It is induced in normal mice by administering cytosine arabinoside (Ara-C) to newborns. Animals are administered Ara-C at a dose of 40 mg/kg body weight daily for the first 3 days after birth (intraperitoneally).	Middle stage: time on the treadmill decreased by 2.9 times. Scores on gait, stance, and hind limb grip tests were on average 4.75 points higher than in control animals. Motor activity decreased by 2.6 times [170].	Early stage: apoptosis of proliferating cells begins. Middle stage: loss of Purkinje neurons and granular cells (decrease in calbindin and NeuN markers). Late stage: high levels of proinflammatory factors: TNF-α, IL-1β, iNOS; decrease in neurotrophic factors (BDNF, GDNF).	0	Park et al. [170]	Not available for sale, reproduces independently.
LPS, mouse	Intracerebellar injection of lipopolysaccharide (LPS) (5 μg/5 μL) to 10-week-old mice.	Middle stage: ataxia-like behavior: significantly reduced coordination of movements, gait abnormalities, and a characteristic “grasping” reflex of the hind limbs [169]. Late stage: abnormal motor behavior (impaired coordination and sluggish hind legs).	Within 1–7 days, there is a sharp increase in the expression of microglial and astrocytic markers (Iba1, GFAP), as well as pro-inflammatory molecules (TNF-α, IL-1β) [169]. In the early days, Purkinje cell apoptosis develops. Chronic inflammation. The cytokine “storm” remains high, reinforcing the pathology.	1	Hong et al. [169]	Not available for sale, reproduces independently.
Ethanol, rat	Liquid diet with alcohol for several weeks	Early stage: After several weeks, coordination disorders and ataxia are observed. Middle stage: Stable motor deficits gradually accumulate: decreased spontaneous activity and slowed movements (bradykinesia), increased delays in balance tests [179]. Late stage: Motor impairments become persistent even with ethanol abstinence. Speculomotor defects (analogous to ataxia) persist.	Histologically, the cerebellum shows noticeable atrophy: a decrease in organ mass and neuron count (decrease in NeuN labeling in the granular and molecular layers) [180]. Chronic reorganization of cerebellar neural networks: increased expression of the Fmr1 gene (Regulator of RNA for neuronal plasticity) and its targets (CREB1, PSD95, mGluR5, NMDA receptors)	0	Dar. [176]	Not available for sale, reproduces independently.
3-acetylpyridine rat	Single subcutaneous/intraperitoneal administration of the neurotoxin 3-acetylpyridine (3-AP)	Early stage: After several weeks, coordination disorders and ataxia are observed. Middle stage: Stable motor deficits gradually accumulate: decreased spontaneous activity and slowed movements (bradykinesia), increased delays in balance tests [179]. Late stage: Motor impairments become persistent even with ethanol abstinence. Speculomotor defects (analogous to ataxia) persist.	Middle stage: There is marked damage to the cerebellar climbing fibers: the cells of the inferior olive degenerate, and signal transmission to the cerebellum is impaired. The amount of glutamate and taurine neurotransmitters in the cerebellum gradually decreases, reflecting metabolic dysfunction. Late stage: The cerebellar neuron deficit stabilizes: the rats’ ataxia persists for a long time (olive atrophy is irreversible). Late stage: The cerebellar neuron deficit stabilizes: the rats’ ataxia persists for a long time (olive atrophy is irreversible). Molecularly: a significant decrease in glutamate and taurine concentrations in the cerebellum and an increase in glutamine in the damaged areas, which corresponds to a prolonged neurotoxic effect.	0	Aghighi et al. [181]	Not available for sale, reproduces independently.
LVV mouse	Lentiviral vector under an enhanced GFAP promoter, selective expression of FLAG-ATXN1^[Q85]^ in Bergmann glia cells (BG). Injection of 3 µL LVV (≈7 × 10^9^ TU/mL) into the cerebellar cortex of P21 WT mice (CD-1 IGS); analysis after ~9 weeks, i.e., at 12 weeks of age.	Early stage (1–2 days): demonstrate a significant reduction in latency on the rotarod compared to the control group that received lentivirus with ATXN1^[Q2]^. The trend toward short-term motor learning persists compared to SCA1 KI [51].	By 12 weeks, LVV-SCA1 mice show reactive astrogliosis in the cerebellar cortex (GFAP and S100β upregulation) together with structural cortical atrophy—thinning of the molecular layer and a reduction in Purkinje cell dendritic arborization (evidenced by decreased Purkinje cell membrane capacitance on whole-cell recordings, interpreted as dendritic collapse). At the synaptic level, PF→PC long-term depression (LTD) is impaired, and the maintenance of depolarization-induced suppression of excitation (DSE) is destabilized/abnormally short-lived.	0	Shuvaev et al. [51]	Not available for sale, reproduces independently.

*: Number of original research articles that explicitly employed the respective model as an experimental object, based on a structured PubMed search using citation analysis and filters described in the Methods Section. ↑: Increased GFAP staining.

**Table 4:** Model selection matrix for SCA1 experimental models.

Model (Species)	Research Goals	Key Readouts	Onset Windows, Time-Course	Key Advantages	Key Limitations
ATXN1^[82Q]^, mouse	1. Rapid discovery 3. Particular mechanisms 8. Cell-type contributions	Fast cerebellar phenotypes: rotarod, dendritic/complex spike metrics, Purkinje firing irregularity and synaptic weakening (e.g., reduced long-term depression) by 3 months, coinciding with ataxia.	Obvious Purkinje cell atrophy beginning at 3 and 4 weeks. Motor function: Rotarod performance declines by 5–6 weeks, balance beam, gait analysis. Electrophysiological hyperexcitability in molecular layer interneurons is measurable by 10–12 weeks, preceding major cell loss.	Fast and cost-effective, large-scale gene modifier and large-scale drug. Suitable for studying Purkinje neuron degeneration and testing therapies targeting the cerebellum isolating Purkinje-centric effects on networks, aiding analysis of electrophysiological biomarkers (simple spike irregularities, etc. Isolates Purkinje neuron contribution to ataxia. It’s ideal for testing interventions aimed at Purkinje cells	No mutant expression outside cerebellum; Very high ATXN1 levels may produce non-physiological effects, no cognitive data, early severe symptoms limiting long-term studies.
Knock-in ATXN1^[78Q/2Q]^, mouse	4. Human relevance 5. Molecular mechanisms 6. Screen for genes	Subtle rotarod deficit starting ~9 months; no overt ataxia. No visible ataxia up. Little Purkinje cell loss or inclusion pathology, mild PC dendritic changes; somatic instability of CAG repeat in tissues.	Phenotype emerges in mid-life (rotarod decline 9–18 months).	Long asymptomatic phase allows testing of stressors or gene knockouts to precipitate or modify disease.	Limited pathology: failed to produce overt ataxia, compensation by normal allele; long duration: requires 9–12 months to observe motor deficits
Knock-in ATXN1^[154Q/2Q]^, mouse	2. Systemic features 4. Human relevance 5. Molecular mechanisms 8. Cell-type contributions	Reproduction of systemic motor incoordination, respiratory phenotype, cognitive dysfunction, etc.	Motor learning deficit on rotarod by 5–6 weeks, memory issues by ~8 weeks, non-motor signs (anxiety, depression-like behavior) manifest by 2–6 months alongside neuropathology. Biochemical and molecular parameters: composition and dynamics of ATXN1 nuclear inclusions, protein (interactome) and RNA (transcriptome, splicing) interaction profiles (innate immune IFN-ISG15 activation by 6–8 weeks, elevated glutamine & creatine on MRS). Neuronal inclusions appear by 6 months, correlating with later-stage degeneration; more robust weight reduction ~32 weeks.	Useful for testing systemic therapies (ASO, gene therapy) that target all affected tissues, reproduces the key manifestations of human disease. Displays molecular changes months before overt ataxia (identification of biomarkers). Long window for intervention: Gradual onset allows testing therapies at pre-, early, and late stages, high human relevance	Slow disease course, behavioral assays (rotarod, mazes) needed for early deficits; subtle phenotypes require large cohorts for drug trials; expanded allele may grow or shrink across generations; limitation of very long-term studies (lifespan ~1 year).
f-ATXN1^[146Q/2Q]^, mouse	2. Systemic features 8. Cell-type contributions 5. Molecular mechanisms 4. Human relevance	Reproduction of systemic motor incoordination, cognitive deficits, wasting with kyphosis, spontaneous respiratory phenotype and decreased survival	Phenotype depends on expression domain. With broad expression (like KI), ataxia and weakness develop by 3–6 months. Motor deficits on rotarod by 6 weeks, impaired locomotor function by 12 weeks, with kyphosis onset and cognitive deficits manifesting by 24 weeks, followed by a hindlimb clasping phenotype and significantly reduced lifespan by 36 weeks.	A platform for trial gene therapy, tissue-specific mutagenesis and assessment of its functional contribution, high human relevance	Breeding complexity (careful genotyping and controls for Cre effects): time-consuming; potentially causing mosaic expression and variability; outcome depends on Cre driver (e.g., Nestin-Cre vs. PC-Cre yields different severity)
ATXN1[^154Q_flox_stop/+]^, mouse	8. Cell-type contributions 5. Molecular mechanisms 7. Drug screens (targeted) 4. Human relevance	Reproduction of motor incoordination (rotarod, open field).	Ubiquitous Cre activation yields ataxia 2 slower than straight KI (onset ~6 months). Quantitative PCR (qPCR) demonstrated a 2-fold increase in ATXN1 mRNA levels in cSCA1 × Sox2-Cre mice (in the cerebellum, brainstem, and spinal cord—and not in skeletal muscle tissue); produced less than half of the pathogenic protein compared with the unmodified SCA1 mice at 3 weeks of age. more robust weight reduction ~28 weeks, decreased distance traveled of mice in the open field (when activated only in specific cells: ChAT-Cre)	Ubiquitous activation recapitulates SCA1-like PC pathology (dendritic atrophy, inclusions) but delayed. Cell-specific contributions toward respiratory failure; Crossing with various Cre lines cleanly separates contributions of neuronal subtypes (e.g., showing Purkinje neurons are necessary for major pathology, motor neurons contribute little to lifespan); An ideal platform to test gene-silencing.	Breeding complexity (Careful genotyping and controls for Cre effects); long monitoring for endpoints (slower phenotypes)
ATXN1^[30Q] D776^, mouse	3. Particular mechanisms 5. Molecular mechanisms 8. Cell-type contributions	Motor discoordination from Purkinje firing abnormalities occurs despite intact neuron count; electrophysiological dysfunction (impaired firing, synaptic alterations).	Impaired development of spinocerebellar connections: reduced climbing fiber translocation along Purkinje cell dendrites and poor synaptic wiring	Isolates the effect of a key pathogenic modification (Ser776 phosphorylation); testing of therapeutics aimed; useful for investigating synaptic plasticity changes; dissect toxic signaling independent of aggregation	Not a full SCA1 model (no polyQ expansion, ataxia without significant Purkinje cell death); limits studies on neuroprotective interventions
ATXN1^[82Q]^, Drosophila melanogaster	1. Rapid discovery 6. Screen for genes 5. Molecular mechanisms 7. Drug screens (initial)	rapid readouts (ocular neurodegeneration, lifespan), formation of nuclear inclusions in retinal photoreceptors).	Drosophila melanogaster: decreased motor activity and life expectancy over several days/weeks.	High-throughput gene screening, fast and cost-effective; short lifecycles, key pathways (Notch, DNA repair, etc.).	Not translate all mammalian complexities (e.g., no Purkinje cells); non-physiological effects are often a consequence of ATXN1 overexpression.
ATXN1^[82Q]^, Danio rerio	1. Rapid discovery 2. Systemic features 7. Drug screens 5. Molecular mechanisms 8. Cell-type contributions	Exploratory behavior	Decreased exploratory behavior correlated with the degree of Purkinje degeneration	High-throughput screening of drugs at the larval stage of fish, short lifecycles, key pathways (Notch, DNA repair, etc.); studying the contribution of individual neurons to cerebellar neurodegeneration phenotypes.	Simpler cerebellar structure, cannot model human cognitive symptoms deeply; Generating stable lines can be time-consuming
Ara-C, mouse	2. Systemic features 7. Drug screens 8. Cell-type contributions	Destroys proliferating cerebellar granular cells; rodents show dysmetria, wide-based gait, impaired rotarod by end of treatment	Selectively ablates dividing cells. Purkinje cells shrink and firing patterns alter, though Purkinje cells shrink and firing patterns alter	Mimics the end-stage effect of ataxias; platform to test anti-inflammatory, neuroprotective and stem cell transplantation agents on motor function; large cohorts can be used without complex genetics	Irreversible cerebellar hypoplasia rather than progressive degeneration; does not modulate ongoing disease; no inclusion bodies or mutant protein
LPS, mouse	2. Systemic features 7. Drug screens (targeting inflammation) 3. Particular mechanisms	Rapid-onset motor deficits (impaired rotarod, ataxic gait, hindlimb clasping); microglial activation	Rapid-onset motor deficits peaking ~1 week post-injection. In cerebellum, robust microglial activation and astrocytosis within 1–7 days; retention time on the rotating rod significantly declined at 4 weeks after LPS injection	Speed and simplicity; large cohorts can be used without complex genetics; suitable for rapid pharmacological testing on ataxia symptoms; dose and location of LPS critically determine outcome	No inclusion bodies or mutant protein; some deficits may recover as inflammation subsides; can confound motor readouts from LPS
Ethanol, rat	3. Particular mechanisms 5. Molecular mechanisms 7. Drug screens	Gradual motor decline (balance and coordination tests worsen over months of exposure: falling off rotarod, widened gait) and an age-dependent rise in blood and CSF neurofilament light (NfL) levels; chronic diet lead to persistent mild ataxia and tremor; broad-based, unsteady gait; enhanced GABA_A signaling, reduced cerebellar metabolic activity; vermis atrophy (selective shrinkage of anterior lobules);	Progressive Purkinje dendritic atrophy and synapse loss develop over 8–10 months on ethanol diet.	Providing insight into mechanisms of Purkinje cell vulnerability (oxidative stress, nutrient deficiency); platform for antioxidant or metabolic therapies; reversible neurotransmitter imbalances	Markers may not be fully SCA1-specific; difficulty in conducting cognitive tests due to ethanol; no inclusion bodies or mutant protein
3-acetylpyridine rat	2. Systemic features 8. Cell-type contributions 7. Drug screens	Decreased spontaneous activity and slowed movements (bradykinesia), increased delays in balance tests; ablates inferior olivary neurons, acute olivo-cerebellar disconnection and subsequent cerebellar cortical atrophy; disrupts NAD synthesis	After several weeks, coordination disorders and ataxia are observed. Stable motor deficits gradually accumulate.	Used to test neuroregenerative approaches (e.g., stem cells, trophic factor delivery) by measuring restoration of motor function; complements genetic SCA1 models by modulating the disruption of the inferior olive-Purkinje cell circuit.	Doesn’t replicate the progressive nature or early subtle deficits of SCA1 (sudden massive cerebellar injury rather than gradual degeneration); no inclusion bodies or mutant protein; most relevant in early post-lesion phase; after that it’s a static deficit model
SCA1 Patient iPSCs	4. Human relevance 5. Molecular mechanisms 7. Drug screens 8. Cell-type contributions	Reduced dendritic branching and smaller cell size compared to isogenic control neurons; transcriptomics, synaptic markers.	SCA1-iPSC-derived Purkinje-like cells show nuclear ATXN1 inclusions over weeks in culture	iPSCs can be guided to different lineages; platform for pharmacological screening; gene editing	Differentiating iPSCs into mature Purkinje-like neurons can take months and yields limited cell numbers, lower survival under stress and altered synaptic connectivity; not as amenable to high-throughput screening as simpler cell lines; incomplete maturity neurons.

## 6. Conclusions

A comprehensive analysis of SCA1 model systems confirms that none of them covers the entire spectrum of pathogenic and clinical manifestations of the disease. Exogenous cell lines (Neuro-2a, HEK293, HeLa, DAOY) allow targeted modification of key cellular and molecular processes—the formation of intranuclear ATXN1 inclusions, disruption of nucleocytoplasmic transport, and rearrangement of protein–protein/RNA interactions—and thus serve as an operational platform for mechanistic testing and high-throughput screening of targeted approaches (ASO, siRNA/shRNA, CRISPR/Cas9). At the same time, the results obtained on these models require mandatory verification on systems with endogenous expression of mutant ATXN1 (SCA1 Patient Fibroblasts, SCA1 Patient iPSCs), which preserve the human genetic context and better reflect relevant phenotypic features.

Analysis of in vivo models demonstrates significant differences in progression rates, tissue selectivity, and response to therapies. These features determine the need for careful selection of an experimental system in accordance with specific research objectives. A comparison of the citation rates of in vivo studies (data presented in Table 2) shows the varying influence of individual model lines on the development of the scientific field and highlights the importance of model accessibility and reproducibility when planning experiments. Thus, transgenic models with high expression are convenient for studying early mechanisms and rapid evaluation of candidates, while knock-in lines with moderate expression are preferable for long-term efficacy and safety; conditional constructs further reveal the tissue-specific contribution of cell populations.

Thus, the sequential use of in vitro and in vivo platforms forms a rigorous preclinical trajectory: from primary screening on cell lines to validation on patient-specific models and subsequent evaluation of efficacy and safety in animal experiments. This multi-level, translation-oriented design increases the predictive value of the results and accelerates their movement toward clinical application in SCA1.

## 7. Future Direction

Despite substantial progress in the characterization and modeling of SCA1, several important challenges remain. Current models, while powerful, each capture only selected aspects of the disease, and no single system fully recapitulates the complexity of human SCA1 physiology. Future research should therefore prioritize the development of more physiologically relevant, accessible, and scalable models that more accurately reflect human-specific pathophysiology, including cell–cell communication, glial contributions, and circuit-level vulnerabilities within an intact tissue context. Advancing induced plu-ripotent stem cell–derived neuronal and glial systems, refining next-generation knock-in and conditional models, and implementing integrative multimodal platforms will be es-sential for bridging the translational gap. Together, these efforts will support more pre-dictive therapeutic testing and accelerate progress toward effective disease-modifying treatments for SCA1.

## Figures and Tables

**Table 1:** Original research articles using in vivo SCA1 models selected for quantitative analysis.

Model: ATXN1^[82Q]^, Mouse		Phrases Quoting the Original Source	Link to Article
№	Article Title		
1	Pre-ataxic loss of intrinsic plasticity and motor learning in a mouse model of SCA1	Here, we sought to investigate the underlying PC pathological events before the onset of ataxia by taking advantage of ATXN1[82Q] mice, a PC-specific mouse model of SCA1.	[28]
2	RNAi or overexpression: alternative therapies for Spinocerebellar Ataxia Type 1	The B05 transgenic mouse model of SCA1 expresses a polyQ expanded human ataxin-1 allele under control of the Purkinje cell specific promoter (Pcp2)	[29]
3	Mesenchymal stem cells ameliorate cerebellar pathology in a mouse model of spinocerebellar ataxia type 1	SCA1-Tg mice (B05 line) on the FVB background [4] were kindly provided by Dr. Harry T. Orr of the University of Minnesota, Minneapolis, MN, USA. SCA1-Tg mice and wild-type (WT) mice with the same genetic background were used for the experiments.	[30]
4	Dendritic potassium channel dysfunction may contribute to dendrite degeneration in spinocerebellar ataxia type 1	ATXN1[82Q] transgenic mice [23] overexpress mutant human ATXN1 with 82 CAG repeats selectively in cerebellar Purkinje neurons under the Purkinje neuron-specific murine Pcp2 (L7) promoter and were maintained on an FVB/NJ background (Jackson Labs).	[31]
5	The insulin-like growth factor pathway is altered in spinocerebellar ataxia type 1 and type 7	we used an SCA1 transgenic (Tg) mouse model (SCA1[82Q] Tg) that expresses a mutant SCA1 allele encoding ATXN1 with 82 glutamines only in PCs (16) and develops a progressive cerebellar degenerative phenotype	[32]
6	Purkinje Cell Expression of a Mutant Allele of SCA1in Transgenic Mice Leads to Disparate Effects on Motor Behaviors, Followed by a Progressive Cerebellar Dysfunction and Histological Alterations	we have described the generation and initial characterization of transgenic animals expressing either a normal humanSCA1 allele (A0− lines; 30 CAG repeats) or a mutant humanSCA1 allele (B0− lines; 82 CAG repeats)	[33]
7	Development of microglia-targeting adeno-associated viral vectors as tools to study microglial behavior in vivo	we chose spinocerebellar ataxia type 1 (SCA1) transgenic (SCA1-Tg) mice that express abnormally expanded ATXN1, specifically in cerebellar PCs under the control of the PC-specific L7 promoter (also known as the B05 line)	[34]
8	ATXN1-CIC Complex Is the Primary Driver of Cerebellar Pathology in Spinocerebellar Ataxia Type 1 through a Gain-of-Function Mechanism	using the Pcp2-ATXN1[82Q] (B05) construct previously described	[25]
9	Early activation of microglia and astrocytes in mouse models of Spinocerebellar Ataxia Type 1	constructs are driven by the Purkinje cell specific (Pcp2) promoter were generated in a FVB/N background and include: (1) the ATXN1[82Q] line (also called the B05 line)	[35]
10	Dysregulation of alternative splicing in spinocerebellar ataxia type 1	mouse models were utilized for described experiments: SCA1 B05 (Pcp2: ATXN1[82Q]) mice	[36]
11	Dopamine D2 Receptor Signaling Modulates Mutant Ataxin-1 S776 Phosphorylation and Aggregation	The SCA1 Tg mice were generated by Drs. Harry Orr and Huda Zoghbi	[37]
12	A cellular system that degrades misfolded proteins and protects against neurodegeneration	To investigate the physiological role of this system, we used a mouse model of SCA1 (B05), which expresses the ATXN1[82Q] transgene (Atxn1tg/−) in the cerebellar Purkinje cells.	[38]
13	In vivo 5-ethynyluridine (EU) labelling detects reduced transcription in Purkinje cell degeneration mouse mutants, but can itself induce neurodegeneration	ATXN1[82Q] transgenic mice were kindly provided by Dr. Harry T. Orr (University of Minnesota, Minneapolis, MN, USA) and were derived from the B05 line that overexpresses human ATXN1 cDNA containing an 82 CAG-repeat under the Purkinje cell-specific Pcp2 promoter (Tg(Pcp2-ATXN1*82Q)5Horr)	[39]
14	A novel function of Ataxin-1 in the modulation of PP2A activity is dysregulated in the spinocerebellar ataxia type 1	transgenic mice carrying the mutant SCA1 allele with 82 CAG repeats (B05) driving the expression in cerebellar PCs of mutant ..Ataxin-1 containing 82 .. glutamines,	[40]
15	Reduction of Protein Kinase A-mediated Phosphorylation of ATXN1-S776 in Purkinje Cells Delays Onset of Ataxia in a SCA1 Mouse Model	Mice generated by our laboratory include ATXN1^[66Q/2Q]^ mice (derived from ATXN1^[78Q/2Q]^ mice), ATXN1−/− null mice [, Pcp2-ATXN1[30Q], and Pcp2-ATXN1[82Q] mice	[41]
16	Aminopyridines Correct Early Dysfunction and Delay Neurodegeneration in a Mouse Model of Spinocerebellar Ataxia Type 1	.. B05 mice express human ataxin-1 with a pathological (82) CAG repeat length specifically within cerebellar PCs using the pcp2 promoter	[42]
17	Dynamic molecular network analysis of iPSC-Purkinje cells differentiation delineates roles of ISG15 in SCA1 at the earliest stage	..we performed iMAD analysis using the public database ..of Pcp2-ATXN1-82Q/2Q transgenic mice (ATXN1-Tg mice)..	[43]
18	Progressive impairment of cerebellar mGluR signalling and its therapeutic potential for cerebellar ataxia in spinocerebellar ataxia type 1 model mice	In most experiments, we used transgenic SCA1 model mice (SCA1-Tg; heterozygous B05 line carrying the human Ataxin-1 gene with an extended 82 glutamine tract under control of the PC-specific L7 promoter) and wild-type (WT) mice of both sexes of the FVB background	[44]
19	Altered Trafficking of Membrane Proteins in Purkinje Cells of SCA1	By overexpressing a full-length SCA1 cDNA-encoding mutant ataxin-1 with 82 glutamines under the direction of the Purkinje cell-specific Pcp2/L7 promoter, we established transgenic mice that develop a progressive ataxia.	[45]
20	Differential effects of Wnt-β-catenin signaling in Purkinje cells and Bergmann glia in spinocerebellar ataxia type 1	..an SCA1 transgenic (SCA1 Tg [82Q]; Pcp2-ATXN182Q/+) (35) line, in which mutant ataxin-1 with 82 glutamine repeats is overexpressed under the PC-specific Pcp2 promoter..	[46]
21	Phosphorylation of ATXN1 at Ser776 in the cerebellum	..crossing homozygous ATXN1[82Q] mice from the B05 line..	[47]
22	Noninvasive Detection of Presymptomatic and Progressive Neurodegeneration in a Mouse Model of Spinocerebellar Ataxia Type 1	SCA1[82Q] transgenic mice (N = 14) that overexpress the mutant human ataxin-1 with an 82 glutamine stretch; these animals belonged to the B05 strain described previously	[48]
23	Dynamics of a Protein Interaction Network Associated to the Aggregation of polyQ-Expanded Ataxin-1	RNA-seq datasets from the cerebellum of three age groups (n = 3 animals per group) of SCA1 B05 transgenic mice (week 5, week 12 and week 28) and age-matched control FVB mice were retrieved from the literature	[49]
24	Cerebellar Transcriptome Profiles of ATXN1 Transgenic Mice Reveal SCA1 Disease Progression and Protection Pathways	Mice utilized had ATXN1 transgene expression directed specifically to PCs using an 850 bp portion of the 5′ upstream region from the Pcp2/L7 gene	[50]
25	Sphingolipid metabolism governs Purkinje cell patterned degeneration in ATXN1[82Q]/+	ATXN1[82Q]/+ mice originated from the transgenic line B05… were kindly provided by Harry T. Orr, Department of Laboratory Medicine and Pathology, Institute for Translational Neuroscience, University of Minnesota, Minneapolis, MN.	[51]
26	Cholecystokinin 1 receptor activation restores normal mTORC1 signaling and is protective to Purkinje cells of SCA mice	One line included mice expressing ATXN1 with an expanded polyQ, ATXN1[82Q], that manifest a progressive disease culminating in Purkinje cell death	[52]
27	Purkinje Cell Ataxin-1 Modulates Climbing Fiber Synaptic Input in Developing and Adult Mouse Cerebellum	… ATXN1[82Q]-S776 (line B05), … mice were used	[53]
28	Altered Capicua expression drives regional Purkinje neuron vulnerability through ion channel gene dysregulation in spinocerebellar ataxia type 1	ATXN1[82Q] transgenic mice … were maintained homozygous for the transgene on an FVB/NJ background.	[54]
**Model: Knock-in ATXN1^[154Q/2Q]^, Mouse**		**Phrases Quoting the Original Source**	**Link to Article**
**№**	**Article Title**		
1	A functional deficiency of TERA/VCP/p97 contributes to impaired DNA damage repair in multiple polyglutamine diseases	We used KI … mutant ATXN1-154Q	[55]
2	miR760 regulates ATXN1 levels via interaction with its 5′ untranslated region	SCA1 mice recapitulate many pathological and behavioral characteristics of SCA1 including motor incoordination beginning at 5 wk of age	[56]
3	Developmental YAPdeltaC determines adult pathology in a model of spinocerebellar ataxia type 1	To investigate the temporal specificity of the potential therapeutic effect of YAPdeltaC, the Tet-ON mice were crossed with mutant heterozygous ATXN1-KI (Sca1154Q/2Q) mice	[57]
4	Assessing the Efficacy of Specific Cerebellomodulatory Drugs for Use as Therapy for Spinocerebellar Ataxia Type 1	SCA1154Q mice were generated by the research laboratories of Drs. Harry Orr and Huda Zoghbi	[58]
5	Indirect Negative Effect of Mutant Ataxin-1 on Short- and Long-Term Synaptic Plasticity in Mouse Models of Spinocerebellar Ataxia Type 1	…and 12-week-old C57BL/6 SCA1 KI mice with 154 CAG repeats in the endogenous locus of ATXN1 gene	[59]
6	Polyglutamine disease toxicity is regulated by Nemo-like kinase in spinocerebellar ataxia type 1	To determine whether Nlk gene dosage affects the SCA1 phenotype, we crossed Nlk heterozygote (NlkRRJ297/+) animals with SCA1 knock-in (ATXN1154Q/+) mice	[60]
7	Mutant Ataxin-1 Inhibits Neural Progenitor Cell Proliferation in SCA1	ATXN1154Q/2Q mice were generated as described	[61]
8	Abnormalities in synaptic dynamics during development in a mouse model of spinocerebellar ataxia type 1	Sca1154Q/2Q mice were kindly provided by Dr. K. Watase at Tokyo Medical and Dental University	[62]
9	Mood alterations in mouse models of Spinocerebellar Ataxia type 1	We first tested mood in ATXN1154Q/2Q knock-in mice, in which one ATXN1 allele has a long CAG expansion (154 repeats) …, while the other allele has 2 CAG repeats which is normal for wild-type mice.	[18]
10	Phosphorylation of ATXN1 at Ser776 in the cerebellum	The generation and genotyping of Sca1154Q/+ mice has been described	[47]
11	Early activation of microglia and astrocytes in mouse models of Spinocerebellar Ataxia Type 1	The Sca1154Q/2Q knock-in line was generated by replacing the mouse ATXN1 with an extended polyglutamine tract in the ATXN1 locus. Originally generated in the C57BL/6J–129/SvEv mixed background	[35]
12	Cellular Fusion for Gene Delivery to SCA1 Affected Purkinje Neurons	Recipients were female, 6–8 week-old, Sca1154Q/2Q knock-in mice; generously provided by the Zoghbi laboratoy, Baylor Medical college/HHMI) and maintained at the University of Florida Animal Care Services.	[63]
13	Altered Capicua expression drives regional Purkinje neuron vulnerability through ion channel gene dysregulation in spinocerebellar ataxia type 1	ATXN1154Q knock-in mice were maintained on a C57Bl6J background (Jackson labs) by crossing ATXN1154Q/2Q males with ATXN12Q/2Q wild-type females.	[54]
14	Stool is a sensitive and noninvasive source of DNA for monitoring expansion in repeat expansion disease mouse models	The generation of the .. SCA1.. mouse models was described previously. .. SCA1 mice (provided by Huda Zoghbi, Howard Hughes Medical Institute, Baylor College of Medicine, Houston, TX, USA)	[64]
15	Dynamic molecular network analysis of iPSC-Purkinje cells differentiation delineates roles of ISG15 in SCA1 at the earliest stage	Therefore, we performed iMAD analysis using the public databases of two SCA1 mouse models, Sca1154Q/2Q knock-in mice (ATXN1-KI mice) .. and examined how the mouse dynamic molecular network is connected with our iPSC dynamic molecular network	[43]
16	VEGF ameliorates the ataxic phenotype in spinocerebellar ataxia type 1 (SCA1) mice	We used tissue from the SCA1 knock-in mice (henceforth SCA1 mice) that express an expanded version of ATXN1 with 154 glutamines, a model that closely mirrors human SCA1	[65]
17	Exercise and Genetic Rescue of SCA1 via the Transcriptional Repressor Capicua	To determine the effects of exercise in SCA1, we implemented a mild exercise regimen in the ATXN1154Q knock-in mice, which bear 154 CAG repeats targeted into the endogenous mouse locus to create a model that recapitulates all aspects of SCA1	[66]
18	Regional rescue of spinocerebellar ataxia type 1 phenotypes by 14-3-3ε haploinsufficiency in mice underscores complex pathogenicity in neurodegeneration	The generation and genotyping of Sca1154Q/+ mice have been described	[67]
19	Differential effects of Wnt-β-catenin signaling in Purkinje cells and Bergmann glia in spinocerebellar ataxia type 1	..of SCA1 were utilized: an SCA1 KI (ATXN1154Q/+) .. strain, which expresses mutant ataxin-1 with 154 glutamine repeats under its endogenous promoter…	[46]
20	A native interactor scaffolds and stabilizes toxic ATAXIN-1 oligomers in SCA1	All mouse procedures were approved by the Institutional Animal Care and Use Committee for Baylor College of Medicine and Affiliates. ATXN1154Q/+ … mice have been previously described	[68]
21	Antisense oligonucleotide–mediated ataxin-1 reduction prolongs survival in SCA1 mice and reveals disease-associated transcriptome profiles	ATXN1154Q/2Q mice, generated by insertion of an expanded CAG repeat into 1 allele of the ATXN1 gene, express ATXN1[154Q] throughout the brain and display 2 key SCA1 phenotypes, ataxia and premature lethality	[69]
22	Ataxin-1 oligomers induce local spread of pathology and decreasing them by passive immunization slows Spinocerebellar ataxia type 1 phenotypes	ATXN1154Q/+..mice have been previously described .. and were backcrossed to C57BL/6 for more than ten generations.	[70]
23	Modulation of ATXN1 S776 phosphorylation reveals the importance of allele-specific targeting in SCA1	ATXN1154Q/2Q (SCA1) mice … and ATXN1–/– (ATXN1-KO) mice (23) were backcrossed to C57BL/6J background for a minimum of 10 generations.	[71]
24	Lithium Therapy Improves Neurological Function and Hippocampal Dendritic Arborization in a Spinocerebellar Ataxia Type 1 Mouse Model	Sca1154Q/2Q mice and their wild-type littermates were obtained from crossings between male Sca1154Q/2Q mice and wild-type female mice on two different backgrounds: mice of C57BL/6J–129/SvEv mixed background; and mice that were obtained after C57BL/6J–129/SvEv mutant mice were backcrossed to C57BL/6J at least five times (>N5 generation).	[72]
25	Hippocampal mitochondrial dysfunction and psychiatric-relevant behavioral deficits in spinocerebellar ataxia 1 mouse model	We used B6.129S-ATXN1tm1Hzo/J knock-in mice (Jackson Laboratory)10 for testing purposes. Heterozygous mice with 154 CAG repeats within exon 8 of the targeted endogenous mouse ATXN1 locus (SCA1154Q/2Q) were used along with homozygous control mice with normal CAG repeats in both ATXN1 loci (SCA12Q/2Q).	[73]
26	Early molecular layer interneuron hyperactivity triggers Purkinje neuron degeneration in SCA1	The ATXN1154Q/2Q knock-in mice (B6.129S-ATXN1tm1Hzo/J), called Sca1 throughout the manuscript… were obtained from Jackson Laboratory.	[74]
27	RAS-MAPK-MSK1 pathway modulates ataxin 1 protein levels and toxicity in SCA1	ATXN1154Q/+ has been previously described.. and has been backcrossed to C57BL/6 for more than ten generations.	[75]
28	Partial Loss of Ataxin-1 Function Contributes to Transcriptional Dysregulation in Spinocerebellar Ataxia Type 1 Pathogenesis	ATXN1154Q/+ and ATXN1 −/− mice have been backcrossed into the C75Bl/6J strain for over ten generations.	[25]
29	Suppression of the novel ER protein Maxer by mutant ataxin-1 in Bergman glia contributes to non-cell-autonomous toxicity	Mutant Atx1-KI mice (B6.129S-ATXN1tm1Hzo; Sca1154Q/2Q), which had been made by one of the co-authors in the Zoghbi’s laboratory, were generous gift from Professor Huda Y Zoghbi (Baylor College of Medicine).	[76]
30	Cerebellar contribution to the cognitive alterations in SCA1: evidence from mouse models	ATXN1154Q/2Q … were gifts from Dr Harry Orr and were backcrossed onto a C57/Bl6 background.	[77]
31	Discovery of Novel Activators of Large-Conductance Calcium-Activated Potassium Channels for the Treatment of Cerebellar Ataxia	In a well characterized and genetically precise model of SCA1, ATXN1154Q/2Q mice	[78]
**Model: f-ATXN1^[146Q/2Q]^, Mouse**		**Phrases Quoting the Original Source**	**Link to Article**
**№**	**Article Title**		
1	An expanded polyglutamine in ATAXIN1 results in a loss-of-function that exacerbates severity of Multiple Sclerosis in an EAE mouse model	f-ATXN1146Q/2Q mice are a conditional knock-in mouse model where the coding exons of one allele of the mouse ATXN1 gene was replaced with the human ATXN1 coding exons using site-specific recombination at flanking FRT and LoxN recombination sites.	[79]
2	Sex Differences in a Novel Mouse Model of Spinocerebellar Ataxia Type 1 (SCA1)	In all experiments, littermate wild-type controls were used when possible. The f-ATXN146Q mice.. were gifts from Dr. Harry Orr and Michael Koob.	[80]
**Model: ATXN1^[30Q]D776^**		**Phrases Quoting the Original Source**	**Link to Article**
**№**	**Article Title**		
1	Early activation of microglia and astrocytes in mouse models of Spinocerebellar Ataxia Type 1	..and the ATXN1[30Q]-D776 line, where replacing the same serine residue with a phosphomimetic aspartate residue causes features of SCA1 to occur even in the absence of a pathogenic polyglutamine tract.	[35]
2	Cholecystokinin 1 receptor activation restores normal mTORC1 signaling and is protective to Purkinje cells of SCA mice	Yet, in contrast to ATXN1[82Q] mice, cerebellar disease in ATXN1[30Q]-D776 mice does not manifest with a progressive cerebellar pathology that cumulates with Purkinje neuron death	[52]
3	Purkinje Cell Ataxin-1 Modulates Climbing Fiber Synaptic Input in Developing and Adult Mouse Cerebellum	In particular, a potentially phospho-mimicking aspartic acid amino acid at position 776 enhances pathogenesis of ATXN1[82Q] and transforms wild-type (Wt) ATXN1[30Q] into a pathogenic protein (ATXN1[30Q]-D776)	[53]
**Model: ATXN1^[82Q]^,** **Drosophila Melanogaster**		**Phrases Quoting the Original Source**	**Link to Article**
**№**	**Article Title**		
1	Lazarillo-related Lipocalins confer long-term protection against type I Spinocerebellar Ataxia degeneration contributing to optimize selective autophagy	In this model of SCA1 … photoreceptors accumulate nuclear inclusions of the human protein and start degenerating during late pupal stage when flies develop at 25 °C.	[81]
2	RAS-MAPK-MSK1 pathway modulates ataxin 1 protein levels and toxicity in SCA1	In parallel, we performed a genetic screen in a Drosophila SCA1 model expressing human ATXN1(82Q) that develops an external eye phenotype in response to ATXN1 toxicity	[75]
3	Cross-species genetic screens identify transglutaminase 5 as a regulator of polyglutamine-expanded ataxin-1	These flies display degeneration of external eye structure in proportion to the expression levels of ATXN1[82Q]	[82]
4	dAtaxin-2 Mediates Expanded Ataxin-1-Induced Neurodegeneration in a Drosophila Model of SCA1	We previously reported an unbiased genetic screen with a Drosophila model of SCA1	[83]
**Model: LPS, Mouse**		**Phrases Quoting the Original Source**	**Link to Article**
**№**	**Article Title**		
1	Acute Inflammation Confers Enhanced Protection against Mycobacterium tuberculosis Infection in Mice	LPS was chosen as a commonly used stimulus to induce acute inflammation in the mouse model	[84]
**Model: Knock-in ATXN1^[78Q/2Q]^,** **Mouse**		**Phrases Quoting the Original Source**	**Link to Article**
**№**	**Article Title**		
1	Cerebellar contribution to the cognitive alterations in SCA1: evidence from mouse models	ATXN178Q/2Q … mice originally on FVB background were gifts from Dr Harry Orr and were backcrossed onto a C57/Bl6 background.	[77]
2	Mood alterations in mouse models of Spinocerebellar Ataxia type 1	.. ATXN178Q/2Q … mice were a gift from the laboratory of Dr. Harry Orr.	[18]
3	Ataxin-1 oligomers induce local spread of pathology and decreasing them by passive immunization slows Spinocerebellar ataxia type 1 phenotypes	..ATXN178Q/+ .. mice have been previously described	[70]

“..” and “…” to indicate pauses in the sentence and to highlight phrases or excerpts from articles that specifically refer to the relevant model.

**Table 2:** In vitro cell line models for spinocerebellar ataxia type 1 (SCA1).

Cell Line, Species	Model Type	Expressed ATXN1 Variant	Source(s)
	Exogenous Models		
HEK293 (Human)	Transient Transfection	ATXN1^[82Q]^	[24,35,62,74,75,76,77,78,79,80,81,82,83,84,85,86,87,88,89,120,130]
HeLa (Human)	Transient Transfection	ATXN1^[85Q]^	[35,38,78,81,82,83]
Neuro-2a (Mouse)	Transient Transfection	ATXN1^[85Q]^	[19,21,24,38,42,56,57,73]
DAOY (Human)	Stable Expression	ATXN1^[82Q]^	[21,29,45,49,84,85]
MSC (Human)	Inducible Tet-On System	ATXN1^[Q82]^	[27,86]
	Endogenous Models		
SCA1 Patient Fibroblasts	Primary Culture	ATXN1^[46Q]^	[87,88,89,90]
SCA1 Patient iPSCs	Reprogrammed iPSCs	ATXN1^[46Q]^	[36,78,87,91,92]

## Data Availability

The data presented in this study are available on request from the corresponding author.

## Abbreviations

The following abbreviations are used in this manuscript:

SCA1	Spinocerebellar ataxia type 1
CAG	cytosine–adenine–guanine
ATXN1	ataxin-1
Ser776	serine 776
ASO	antisense oligonucleotide
RNAi	RNA interference
NfL	neurofilament light protein
iPSC	induced pluripotent stem cell
NLS	nuclear localization signal
UPS	ubiquitin–proteasome system
ATXN1-CIC	ataxin-1-Capicua
CIC	Capicua
HMGB ½	High-Mobility Group Box ½
LANP	leucine-rich acidic nuclear protein
PKA	protein kinase
RBM17	RNA-Binding Motif Protein 17
U2AF65	U2 snRNP auxiliary factor 65 kDa
NLK	Nemo-like kinase
ISG15	Interferon-stimulated gene 15
GFI1	growth factor independence 1
SMRT	Silencing Mediator of Retinoid and Thyroid Receptors
Tip60	Tat-interacting protein 60
RORα	retinoid-related orphan receptor α
Boat1	ATXN1-like paralog
RPA	replication protein A
ASR	acoustic startle response
FST	forced swim test
CaB	calbindin
PV	Parvalbumin
CCK	cholecystokinin
LPS	lipopolysaccharide
Ara-C	Cytarabine
